# Unearthing the hidden world of roots: Root biomass and architecture differ among species within the same guild

**DOI:** 10.1371/journal.pone.0185934

**Published:** 2017-10-12

**Authors:** Katherine Sinacore, Jefferson Scott Hall, Catherine Potvin, Alejandro A. Royo, Mark J. Ducey, Mark S. Ashton

**Affiliations:** 1 ForestGEO, Agua Salud Project, Smithsonian Tropical Research Institute, Apartado, Balboa, Ancón, Panamá, Panamá; 2 Department of Natural Resources and the Environment, University of New Hampshire, Durham, New Hampshire, United States of America; 3 Department of Biology, McGill University, Montreal, Québec, Canada; 4 Forest Service, Northern Research Station, Forestry Sciences Laboratory, Irvine, Pennsylvania, United States of America; 5 School of Forestry and Environmental Studies, Yale University, New Haven, Connecticut, United States of America; Kerala Forest Research Institute, INDIA

## Abstract

The potential benefits of planting trees have generated significant interest with respect to sequestering carbon and restoring other forest based ecosystem services. Reliable estimates of carbon stocks are pivotal for understanding the global carbon balance and for promoting initiatives to mitigate CO_2_ emissions through forest management. There are numerous studies employing allometric regression models that convert inventory into aboveground biomass (AGB) and carbon (C). Yet the majority of allometric regression models do not consider the root system nor do these equations provide detail on the architecture and shape of different species. The root system is a vital piece toward understanding the hidden form and function roots play in carbon accumulation, nutrient and plant water uptake, and groundwater infiltration. Work that estimates C in forests as well as models that are used to better understand the hydrologic function of trees need better characterization of tree roots. We harvested 40 trees of six different species, including their roots down to 2 mm in diameter and created species-specific and multi-species models to calculate aboveground (AGB), coarse root belowground biomass (BGB), and total biomass (TB). We also explore the relationship between crown structure and root structure. We found that BGB contributes ~27.6% of a tree’s TB, lateral roots extend over 1.25 times the distance of crown extent, root allocation patterns varied among species, and that AGB is a strong predictor of TB. These findings highlight the potential importance of including the root system in C estimates and lend important insights into the function roots play in water cycling.

## Introduction

In recent decades there has been a growing interest in increasing tree cover on deforested and degraded lands to improve agricultural production [1–3], restore biodiversity values [4,5], provide timber revenue [6–8], improve carbon sequestration [9], and generate or protect water related ecosystem services [10]. Actively planting trees has received significant attention for the role it can play in enhancing ecosystem services [11–14]. Perhaps the most studied aspect of tree planting relates to carbon sequestration [15–18] as it provides a unique opportunity to combat rising CO_2_ levels, an important step toward climate regulation. Carbon (C) assimilation estimates in reforested areas are critical and will hinge on accurate characterization of C stores and net primary productivity (NPP) [19,20].

Estimates of C stores or NPP are typically based on biomass equations. The most common methods to determine aboveground biomass (AGB) of forests include forest inventories combined with allometric tree biomass regression models or airborne and satellite-based remote-sensing techniques [21–24]. Remote sensing techniques such as LiDAR allow for efficient and accurate biomass estimates in forests. However, accurate estimates in plantations can be more challenging with LiDAR due to a less stratified canopy layer [25,26]. For this reason, field-based inventory methods to calculate biomass in plantations still heavily rely on adoption of species-specific biomass equations that typically use basal diameter (BD) or diameter at breast height (DBH) to calculate individual-tree biomass [27–30].

Major efforts to compile accurate biomass estimates include the GlobAllomeTree (globallometree.org), a database for sharing biomass equations. Biomass equations to estimate AGB are abundant [31–33], yet there are few that include belowground biomass (BGB) estimates as well [19,34–36]. This is a major source of error when calculating forest C stocks [37,38]. The fieldwork to assess belowground and aboveground biomass can be prohibitively costly, time consuming, and labor-intensive because it involves excavation of entire root systems [39]. As a result excavation studies are scarce [40]. For this reason, many studies use indirect measures including soil cores that may or may not be accompanied by the use of DNA barcodes to sample BGB and identify root species [39,40]. Although non-destructive methods for assessing biomass of both above and below ground are important, they have the potential to under- or overestimate the underground component [41]. Even destructive methods inherently underestimate the belowground component. This is partly because roots break easily during the excavation process or the soil is not sufficiently excavated to expose the entire root system. Fine roots are typically excluded from the calculations due to their fragility such that studies excluding fine roots—independent of the root diameter threshold for distinguishing between fine and coarse roots—will underestimate the total root biomass.

Challenges of excavations often relegate studies to assessing biomass at the nursery stage of tree growth, where the excavation and weighing of small trees is much more feasible [42]. However, a potential downside with relying on biomass equations from nursery studies is the potential for error when scaling from seedling and sapling to larger trees and forests [43], especially since trees growing in homogenous, nutrient rich, well-watered nurseries might behave differently than those in field conditions [35].

Better predictions of C sequestration in plantations require calculating both above- and below-ground biomass accurately, for a wide range of species and conditions. Accurate characterization of total tree biomass is critical for robust estimates of C storage [37,38]. Root excavation work and mapping of the root system also provide an opportunity to enhance hydrological models that aim to understand the flow of water in forested systems. Rooting depth, distance, and volumes are critical but missing links to predicting water update by trees [44–49].

Here we not only provide models to estimate BGB, AGB, and TB, but provide novel and informative data describing root architecture in their hidden world. In this study, we create models based on planted 6-8-year-old trees in Panama. We also map root structure—both horizontal and vertical distances, depths, and direction. We ask the following questions:

1. What is the relative allocation to belowground biomass of the study trees and how does this inform our understanding of carbon sequestration in plantations (and forests)?
2. Does aboveground biomass correlate strongly with total biomass?
3. How does the aboveground biomass to belowground biomass ratio vary by species?
4. How does the proportional allocation of leaves, branches, stems, and roots vary by species?
5. How does root architecture vary by species? And how does this relate to previous notions of belowground structure and root foraging?

## Materials and methods

### Study sites

Our research sites are located within two plantations in Panama—Sardinilla (9°19’ N, 79°38’ W, 70 m a.s.l.) and in Soberanía National Park (9°13’ N, 79°47’ W, 330 m a.s.l). The Soberania National Park site is managed under a Memorandum of Understanding between the Ministry of the Environment (formerly, and at the time of the research, the National Environmental Authority (ANAM)) and the Smithsonian Tropical Research Institute by the Smithsonian Tropical Research Institute for the period covering 2000 to 2020 for the explicit purpose of conducting reforestation and restoration research with native species. The Sardinilla site is rented by the Smithsonian Tropical Research Institute for reforestation and restoration research and managed by Co Author Potvin. Both land owners gave permission under the signed agreements. Research was conducted at both sites under the research permits issued by the Ministry of the Environment (formerly ANAM) to Co-authors Hall and Potvin where all individuals involved in the work were included in their permits.

Trees were planted between 2001 and 2003 in 3 m by 3 m spacing. At two years of age, the Soberanía sites were thinned by 50% to allow uninhibited aboveground growth. Spacing at harvest and excavation varied by tree but tree spacing was an average of 4.6 m (minimum of 1.4 m and maximum of 15.0 m). Both study sites receive an average of 2300 mm annual rainfall and have a four-month dry season when there is less than 100 mm of rainfall between January and April. Mean daily maximum and minimum temperatures are 32 and 23°C respectively [31]. During the wet season of 2009, 40 trees were harvested to estimate both below- and aboveground biomass. Six different species are represented in this dataset. For more information on plantation design see Coll et al. 2008 [35] (Sardinilla) and van Breugel et al. 2011 [31] (Soberanía).

### Field data

#### Sampling of leaves, branches, and trunks

In 2009, six or seven trees per species (*Anacardium excelsum*, *Cedrela odorata*, *Dalbergia retusa*, *Pachira quinata*, *Tabebuia rosea*, and *Terminalia amazonia*) were excavated for a total of 40 trees [50]. These tree species were selected because they comprised of a broad range of phenological, architectural, and physiological traits. However, the majority of the selected species represent long-lived pioneers. The exception is *Dalbergia retusa*, which is shade tolerant but is successful in high light conditions [51]. They are also known to grow well in the climate and soils of the study sites. Further, all of them are fast-growing species that are of economic importance [52,53]. Trees were excavated when they were fully leaved since some of the species are deciduous.

Trees between 6 and 8 years old were individually selected and marked for excavation. Once trees were selected, the diameter at breast height (diameter of tree bole 1.3 m above soil surface), basal diameter (diameter of bole 10 cm above soil surface or the buttress if present), tree height, and crown diameter (outer most living branch in the north-south and east-west directions) were measured. For the species studied herein, only two species, *T*. *amazonia* and *C*. *odorata* are known to have distinct buttresses. While the basal diameter was measured just above the buttress, all sections of the tree were still included in biomass estimates. Next, all leaves of an individual tree were harvested, placed in a paper bag, and weighed in the field immediately. Leaves were then sub-sampled and oven-dried at 70°C for three days. Next, branches were identified and divided into two subgroups: Primary branches (branches that connect directly to the bole of the tree) and secondary branches (branches that connect to primary branches). Primary and secondary branches were weighed in the field immediately after harvest and then sub-sampled and dried to constant weight. A dry fraction was calculated and used to calculate dry weight of branches.

After the leaves and branches were removed, the trunk was cut into three sections: lower, middle, and upper. The trunk was cut into sections so that the weight would be under the limit of the field scales. The length of each trunk section was measured and weighed and then sub-sampled and dried as the branches were.

#### Sampling of roots

**Fig 1:**
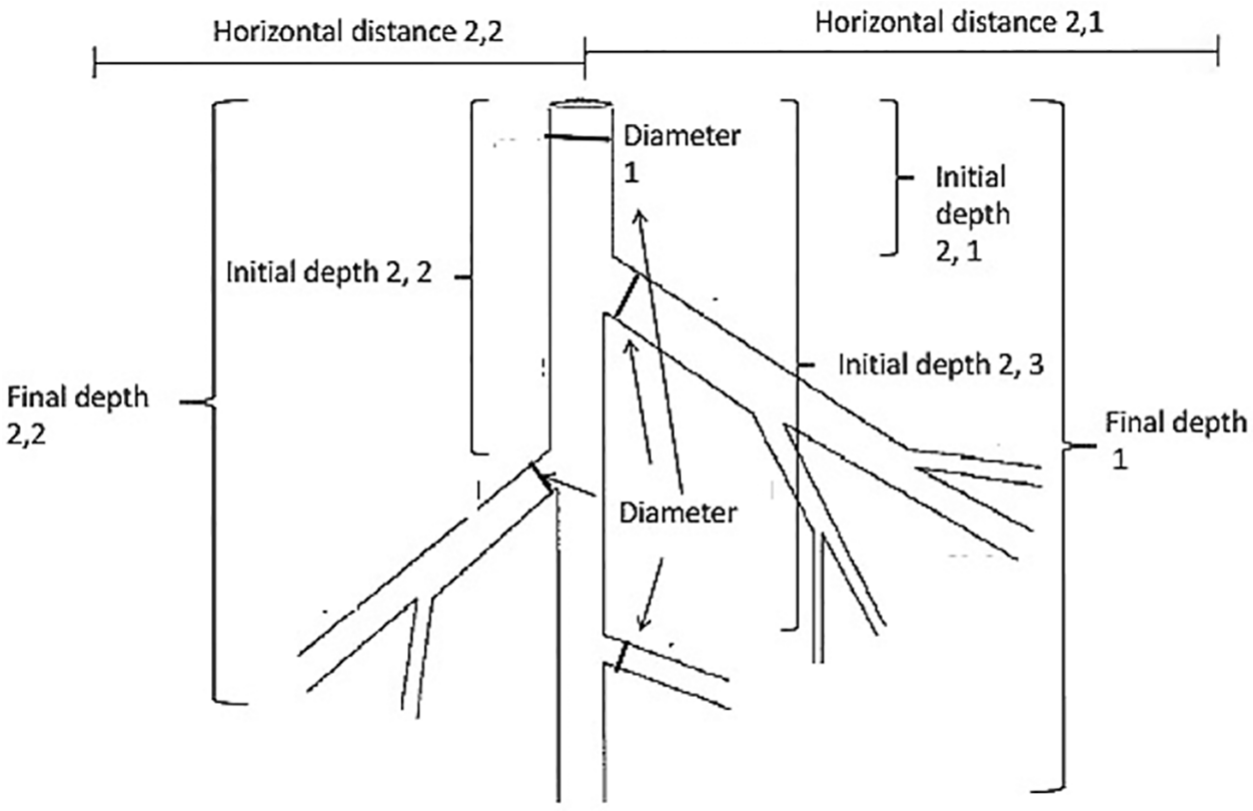
Diagram of root sampling. Diagram of root components measured during excavation process. The main (primary) root was identified as the root that traveled perpendicular to the soil surface (labeled: 1), directly below where the bole of the tree connects (at the top of the diagram). The total depth of the main root was measured from the point of origin to the final depth, which was determined as the point where the diameter of the root was 2 mm. The diameter of the main root was taken at the point of origin. Secondary roots (labeled: 2,1; 2,2; 2,3) were identified as roots originating from the main root. The diameter of the secondary roots was taken at the point where the secondary root connected to the main root. The horizontal distance that secondary roots traveled was measured perpendicular to the soil surface (labeled: Distance 2,1; Distance 2,2). The initial depth of all secondary roots was measured as the vertical depth from the soil surface to the point of origin on the main root. The final depth of all secondary roots was measured as the vertical depth from the soil surface until the point where the diameter of the secondary root reached 2 mm.

Prior to root excavation, the area around the bole of the tree was cleared of litter and fallen branches from neighboring trees to have better access to the roots. A tarp was then placed as a roof over the area to be excavated to prevent rainwater and the associated erosion from entering the pits. To prevent excessive breaking of the roots, a maximum of four to five people excavated each tree. In the cases where roots were broken during the excavation, they were placed in a bag labeled “broken roots” and subsequently cleaned and weighed like any other root section. To excavate soil, small shovels and rakes were used to slowly remove soil from around the area closest to the bole of the tree. As soil was being removed, small pointed sticks (0.5 cm in diameter and about the length of a pencil) were used to remove soil around the roots. Soil being removed was transported in buckets to an area away from the excavation, as to not interfere with the exposed roots. The total quantity of soil removed from each tree excavation was not recorded. Heavy rain events during the excavation brought soil back into the excavation pit and would have added bias to the soil weight calculations. However, using the location (depth below soil surface and horizontal distance from tree bole) of the tips (end of each root), we calculated the rooting volume. The rooting volume, or volume of soil removal, ranged between 1.03 m^3^ and 418.9 m^3^ per tree. The vertical depth and horizontal distance that was excavated defined the size of the tree’s rooting zone. All trees were excavated just beyond the deepest and farthest point of each root excavated. These values ranged from the soil surface (0.0 m) to 3.5 m in depth and distances from the bole of the tree (0.0 m) to 20.5 m (more detail on depths and distances in S3 Table). The area was determined to be sufficiently excavated when all coarse roots ≥ 2 mm in diameter were exposed and visible. Roots below 2 mm in diameter were not excavated, as this is the generally accepted cut-off between coarse and fine roots. Given the high clay content of the soils, it was not possible to harvest the fine roots without severe damage [35]. After all the coarse roots were exposed, all roots were identified and marked with a unique tag number. Main (or primary) roots and secondary roots were identified using the same procedure done with the branches (Fig 1). Only one tree (a *Cedrela odorata*) did not have a distinct main root. While all other trees in the dataset had a distinct main root, 6 individuals from four species had 2 or more main roots. The diameter of the main (or primary) root where the root originates and the final depth were recorded. The diameter of the secondary roots, the initial depth of the secondary roots (the depth below the soil surface where the root initiates from the main root), and the final depth of the roots (the depth below the soil surface where the root tip ends) were recorded. Next, the distance the root traveled horizontally and vertically was measured. Finally, individually marked roots were cut and weighed and processed in the field and laboratory using the same method as for the branches and bole. A final dry weight was taken for each individually marked primary and secondary root after the roots were placed in the oven for at least 5 days and had reached constant weight.

### Biomass calculations and comparisons

We fitted a species-specific allometric regression equation ln(y) = a + b*ln(x) where y is the natural log of aboveground biomass (AGB), the natural log of belowground biomass (BGB), or the natural log of total biomass (TB) and x is the natural log of diameter at breast height (DBH) or the natural log of basal diameter (BD). Each species-specific equation was based on 6–7 trees while the multi-species equations were based on 40 trees with DBH ranging from 2.0 cm to 35.0 cm (Table 1).

**Table 1:** Summary data for harvested species. Site: number of trees excavated in Sardinilla and Soberania. Harvest: number of trees harvested in total for each species. DBH_ave_: average diameter at breast height in cm. DBH_min_: minimum diameter at breast height in cm. DBH_max_: maximum diameter at breast height in cm. Height_ave_: average height of tree in meters (m).

Species	Site		Harvest				
	Sardinilla	Soberania	#	DBH_ave_	DBH_min_	DBH_max_	Height_ave_
*Anacardium excelsum*	7	-	7	15.7	11.4	21.8	9.3
*Cedrela odorata*	6	-	6	22.2	12.4	27.6	14.8
*Dalbergia retusa*	2	5	7	7.2	2	11.6	5.9
*Pachira quinata*	3	3	6	15.8	13.8	20.8	8.2
*Tabebuia rosea*	7	-	7	16.5	8.2	35	8.4
*Terminalia amazonia*	1	6	7	10	6.1	15.9	7.9

Here, we report models that predict AGB, BGB, TB using either DBH or BD as predictors. We also ran models where stem biomass (SB), branch biomass (BB), and foliar biomass (FB) were predicted. Originally, we also used height (H) and a combination of H with DBH or BD as predictors (S3 Table), but H reduced the strength of predicting biomass, likely due to the small sample size of our study. We compared linear models, power models, and polynomial models, selecting the best model based on AIC. To evaluate the our fitted allometric regression models, we used (i) the proportion of variance explained by the model (R^2^ adjusted for the number of predictor variables); (ii) the mean squared error (RMSE); (iii) the mean of the absolute relative differences between the model biomass estimates and the observed biomass values (%); and (iv) the Akaike information criterion (AICc). Here we report species-specific models for AGB, BGB, and TB based on DBH and BD as (i) these predictors often best explained biomass estimates and (ii) AGB, BGB, and TB are most commonly used for carbon stock estimates. We also include a multi-species model that predicts AGB, BGB, and TB using DBH or BD and either a combination of DBH or BD with wood specific gravity (WSG). Finally, ran a linear regression comparing AGB to TB, BGB to TB, and AGB to BGB, determining significance with the maximum likelihood estimator.

### Biomass components

We calculated the fraction of belowground biomass to aboveground biomass components for all species. Aboveground biomass components included the biomass of leaves, branches, and stems while the belowground biomass included the roots. We used a MANOVA on log-transformed data to compare variance among species allocation to leaf, branches, stems, or roots, and a post-hoc Tukey test to test for differences among the species and their components.

### Root architecture

From the root diagrams, we calculated root structure estimates (including rooting depth, rooting horizontal distance, convex root area, and rooting volume) by species and scaled them to diameter at breast height. We scaled the measurements by basal diameter to have more accurate comparisons among species and trees of different sizes. Scaling by BD (or DBH) is a common method used in the forestry [54,55] and plant physiology [56–58] literature to compare traits among trees of different sizes. Diameter at breast height typically scales linearly to the tree crown [59,60], and we hypothesized this relationship this relationship for the roots as well. We calculated maximum and mean rooting depth by BD for all species, as well as maximum and mean horizontal rooting distance (m) by BD for all species. Additionally, we calculated effective system radius (m), which is the square root of the convex area divided by pi. To calculate convex rooting area, roots were assumed to travel exactly on the line indicated by the direction in the data; for example, a root with direction northeast was assumed to travel exactly on a bearing of 45 degrees. We calculated rooting volume (m^3^) by taking the coordinates of the root “tips” (the final rooting point based on distance from either the main or taproot) and treating them as the outer points of an upside-down umbrella, with the taproot or main root depth as the point of the umbrella. Main or taproots were operationally defined as the root that extended downward from the bole of the tree, hereafter referred to as main root. Rooting volume was calculated as the volume of the three-dimensional convex hull of the root tips, plus their projection onto the horizontal plane at the elevation of the root collar. Finally, we calculated the ratio of main or taproot dry weight to total root dry weight for all trees. A ratio closer to 1 suggests the tree allocates most of their total belowground biomass to a main root. A ratio that approaches zero suggests that the tree allocates more resources to lateral roots, or to a rooting architecture that lacks a clear central main root.

To assess species differences for mean and maximum rooting distance, mean and maximum rooting depth, and convex volume we used ANOVA. Post-hoc Tukey tests were used to test if differences were significant among species for rooting distance, rooting depth, and convex volume. We also used beta regression with a maximum likelihood estimator to compare BD to %BGB (Eqs 1 and 2). Beta regression models proportions directly, without a need for transformation of those proportions. The basic beta regression model is:

y~β(μ,Φ) (1)

where Φ is a precision parameter, and μ is a function of the predictor variables, i.e.

$$\mu =\text{logistic}(\Sigma_{j}\;\beta_{j}\;x_{j})$$ (2)

To assess the ratio of main root to total root weight among species, we also used beta regression with a maximum likelihood estimator [61]. We also compared mean root system radius (mean distance roots traveled from bole of tree) and mean crown radius (mean distance crown branches traveled from bole of tree) using a linear model and likelihood ratio test.

## Results

### Species-specific allometric models

**Fig 2:**
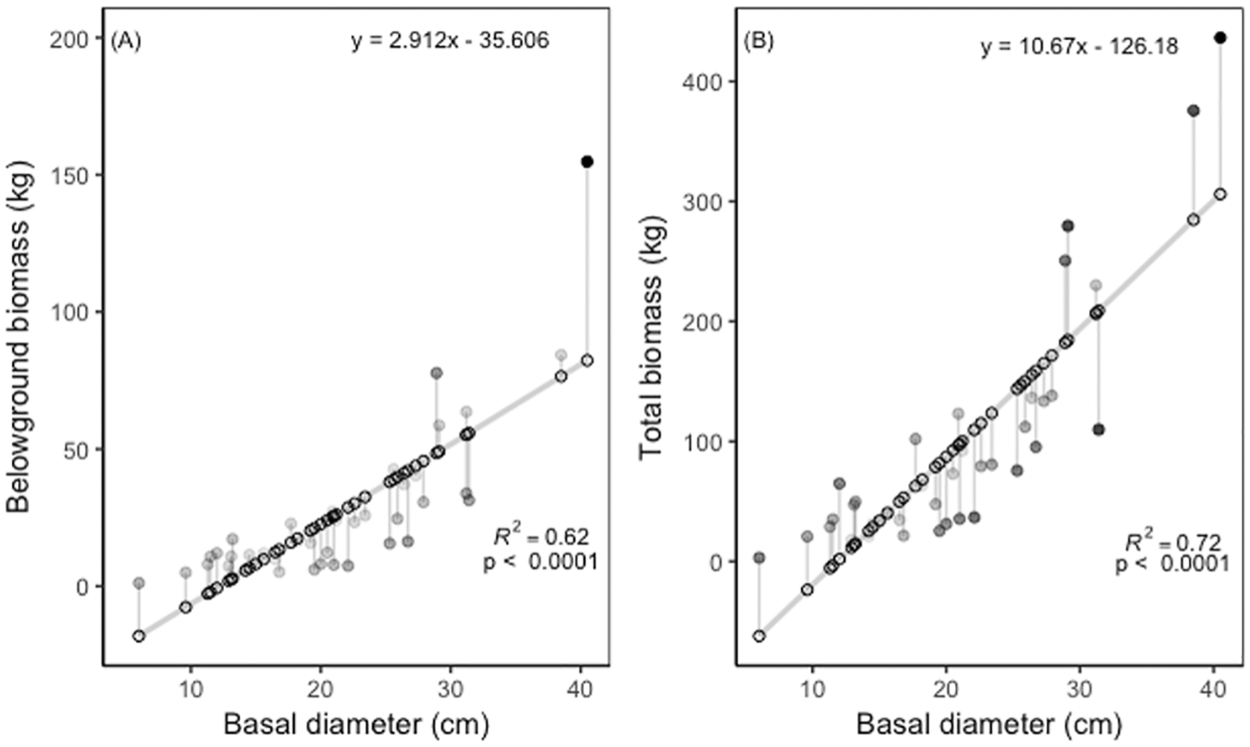
(A) Belowground biomass (kg) and (B) total biomass (kg) for six study species by basal diameter (cm). Belowground biomass by basal diameter when species are pooled (R^2^_adj_, 0.62, p < 0.0001). Total (below- and above-ground biomass) by basal diameter when species are pooled (R^2^_adj_, 0.72, p = 0.0001). Open circles represented modeled predicted values. Filled circles represent the residuals with darker circles being further from the predicted values than lighter, gray shaded circles. Gray line represents the linear model.

**Fig 3:**
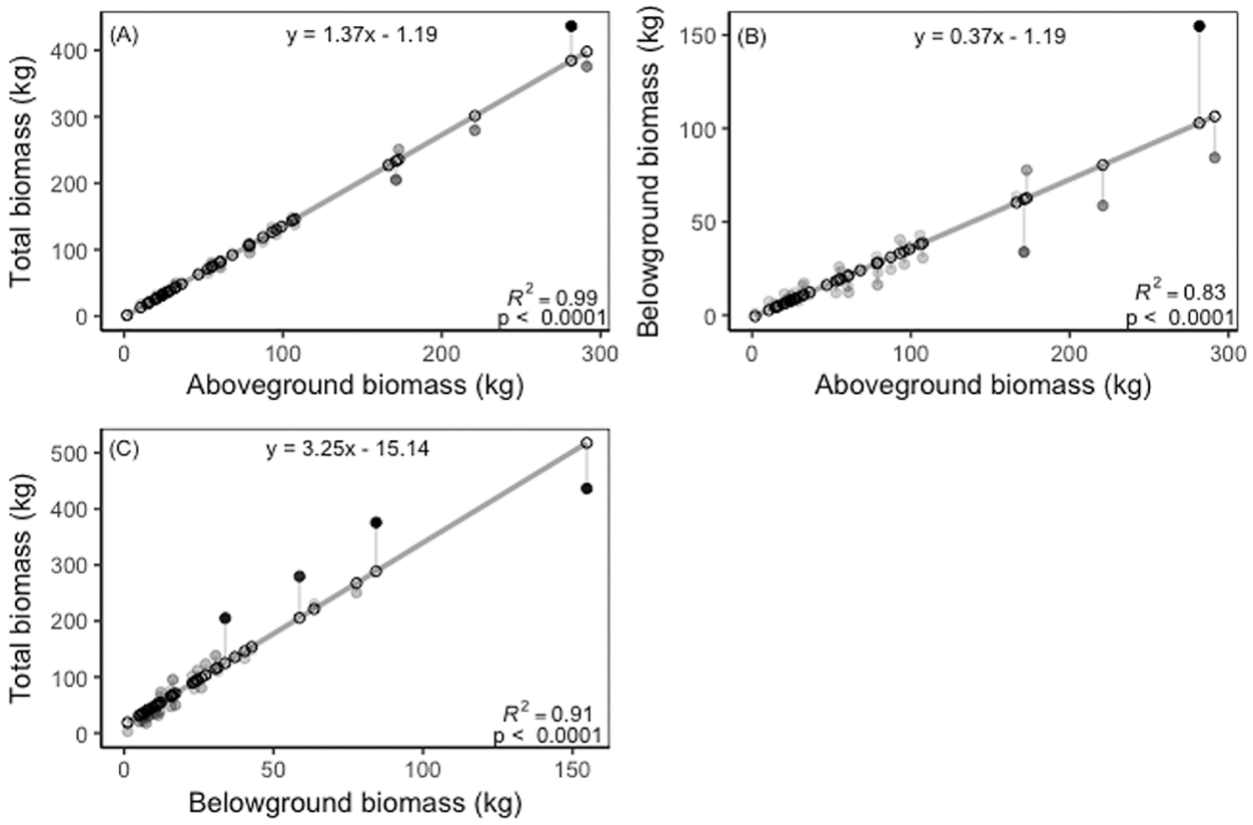
(A) Aboveground biomass (kg) versus total biomass (kg), (B) aboveground biomass (kg) versus belowground biomass (kg), and (C) belowground biomass versus total biomass of pooled species. Open circles represented modeled predicted values. Filled circles represent the residuals with darker circles being further from the predicted values than lighter, gray shaded circles. Gray line represents the linear model.

Our species-specific aboveground biomass allometric models explained between 70.8 and 98.0% of the variance in AGB. We found that for AGB predictions, BD was typically a stronger predictor than DBH (Table 2). BD had an explanatory power between 82.3 and 98.0% while DBH explained between 70.8 and 96.7% of the variation in AGB. The only species where DBH was a stronger predictor for AGB was with *T*. *rosea*. The belowground biomass allometric models explained between 67.8 and 95.2% of the variance in BGB. Here, we found again that BD was typically a better predictor than DBH (Table 2; S2 and S3 Tables for more details). The exception to this rule was for both *C*. *odorata* and *T*. *rosea*, where DBH was a stronger predictor of BGB than BD. For our multi-species model using WSG and DBH, we found that the explanatory power of the regression was highest for TB (R^2^_adj_, 0.816), followed by AGB (R^2^_adj_, 0.810) and BGB (R^2^_adj_, 0.785) (Table 3). There was also a strong relationship between BD and TB (R^2^_adj_, 0.72, p < 0.0001) and with BD and BGB (R^2^_adj_, 0.62, p < 0.0001), with *T*. *rosea* having the greatest TB compared to the other species (Fig 2). As expected, we also found that AGB was strongly correlated with TB (R^2^_adj_, 0.99, p < 0.0001 (Fig 3). Finally, we also found a strong relationship between AGB and BGB (R^2^_adj_, 0.83, p < 0.0001) and between BGB and TB (R^2^_adj_, 0.91, p < 0.0001) (Fig 3).

**Table 2:** Species information for 6 species used to construct regression models. Aboveground biomass: models for aboveground biomass. Belowground biomass: models to predict belowground biomass. Total biomass: models to predict total above and belowground biomass. Left column uses DBH (diameter at breast height, in cm) to predict biomass. Right column uses BD (basal diameter, in cm) to predict biomass. Models: ‘*a*’ and ‘*b*’, coefficients for the species-specific allometric regression models in ln(y) = a + b x ln(x), where y is either AGB, BGB, or TB and x is either DBH or BD. R^2^, the adjusted R^2^; RMSE, root mean squared error; AICc, the second-order Akaike’s information criterion.

	ABOVEGROUND BIOMASS									
Species	DBH					BD				
	*a*	*b*	R^2^	AICc	RMSE	*a*	*b*	R^2^	AICc	RMSE
*Anacardium excelsum*	-1.530	2.105	0.708	16.43	0.341	-3.290	2.411	0.946	4.65	0.147
*Cedrela odorata*	-4.169	2.900	0.959	11.46	0.172	-5.031	2.963	0.972	9.03	0.140
*Dalbergia retusa*	-1.229	2.400	0.839	24.15	0.591	-4.739	2.959	0.943	16.89	0.352
*Pachira quinata*	-5.700	3.360	0.823	14.07	0.235	-9.910	4.335	0.980	2.12	0.079
*Tabebuia rosea*	-2.349	2.309	0.967	9.22	0.203	-4.926	2.809	0.955	11.31	0.236
*Terminalia amazonia*	-0.262	1.735	0.744	16.77	0.349	-2.363	2.321	0.823	14.19	0.290
	BELOWGROUND BIOMASS									
Species	DBH					BD				
	*a*	*b*	R^2^	AICc	RMSE	*a*	*b*	R^2^	AICc	RMSE
*Anacardium excelsum*	-2.004	1.821	0.846	8.91	0.199	-2.862	1.872	0.864	8.08	0.188
*Cedrela odorata*	-4.572	2.716	0.952	11.56	0.173	-5.062	2.679	0.883	13.89	0.271
*Dalbergia retusa*	-1.305	2.010	0.868	20.07	0.442	-4.048	2.401	0.904	17.87	0.378
*Pachira quinata*	-3.967	2.259	0.628	16.12	0.254	-7.284	3.071	0.868	9.93	0.151
*Tabebuia rosea*	-3.139	2.321	0.944	13.08	0.268	-5.353	2.703	0.838	20.50	0.455
*Terminalia amazonia*	-0.701	1.416	0.678	14.34	0.331	-2.812	2.048	0.912	6.99	0.173
	TOTAL BIOMASS									
Species	DBH					BD				
	*a*	*b*	R^2^	AICc	RMSE	*a*	*b*	R^2^	AICc	RMSE
*Anacardium excelsum*	-1.094	2.040	0.759	14.24	0.291	-2.665	2.293	0.966	0.49	0.109
*Cedrela odorata*	-3.705	2.854	0.963	10.63	0.160	-4.471	2.891	0.956	11.65	0.175
*Dalbergia retusa*	-0.625	2.279	0.845	23.11	0.549	-3.907	2.791	0.935	17.06	0.356
*Pachira quinata*	-4.827	3.131	0.799	15.28	0.236	-8.838	4.068	0.969	4.03	0.093
*Tabebuia rosea*	-1.942	2.302	0.973	7.77	0.183	-4.386	2.761	0.927	14.49	0.297
*Terminalia amazonia*	0.203	1.651	0.738	16.28	0.337	-1.902	2.250	0.854	12.19	0.252

**Table 3:** Multi-species model using wood specific gravity. Multi-species aboveground biomass (AGB), belowground biomass (BGB), and total biomass (TB) model using six study species. Left panel shows models using diameter at breast height (DBH; cm) and wood specific gravity (WSG) and left panel shows models using basal diameter (BD; cm) and WSG. Models: ‘*a*’, ‘*b*’, and ‘*c*’, coefficients for the species-specific allometric regression models in ln(y) = a + b x ln(x) + c x ln(z), where y is either AGB, BGB, or TB and x is either DBH or BD, and z is WSG. R^2^, the adjusted R^2^; RMSE, root mean squared error; AICc, the second-order Akaike’s information criterion.

MULTI-SPECIES MODELS												
Output		DBH + WSG						BD + WSG				
	*a*	*B*	*c*	R^2^	AICc	RMSE	*a*	*b*	*c*	R^2^	AICc	RMSE
AGB	-0.670	2.238	1.919	0.842	46.77	0.403	-3.070	2.496	0.827	0.792	57.68	0.462
BGB	-1.267	2.139	2.223	0.845	39.65	0.368	-3.524	2.370	1.168	0.782	53.24	0.437
TB	-0.225	2.204	1.992	0.854	40.94	0.374	-2.586	2.456	0.915	0.803	53.10	0.436

### Biomass allocation

#### Belowground versus aboveground biomass

**Fig 4:**
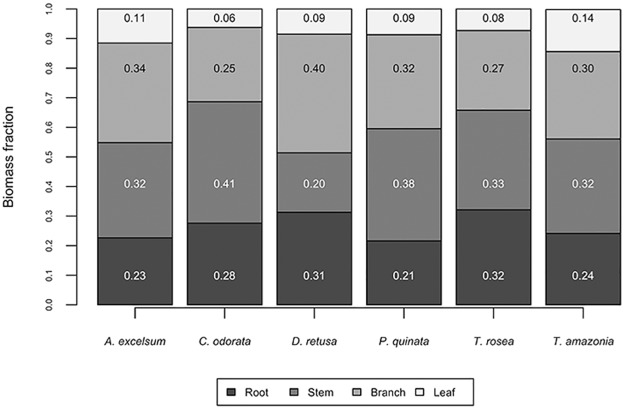
Biomass fraction with tree components. Biomass fraction of root, stem, branch, and leaf components of 6 study species.

**Fig 5:**
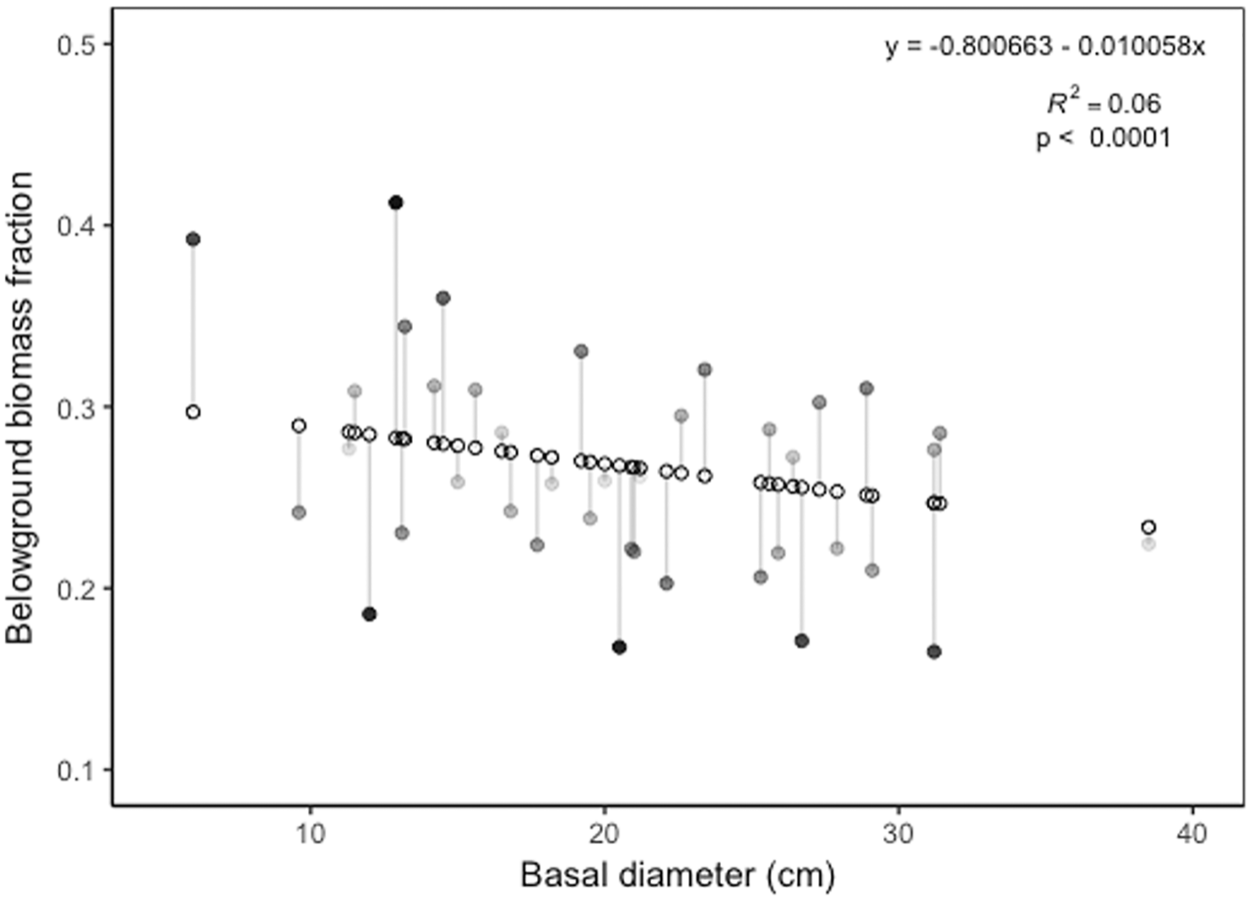
Basal diameter (BD) by % belowground biomass (%BGB). The relationship between BD and %BGB for all six study species based on beta regression and likelihood ratio test (pseudo R^2^_adj_ = 0.06, χ^2^ = 2.6036, p < 0.0001). Open circles represented modeled predicted values. Filled circles represent the residuals with darker circles being further from the predicted values than lighter, gray shaded circles.

Belowground biomass (BGB) varied by species and accounted for between 21% and 32% of the total tree biomass, with an average belowground fraction (%BGB) of 26.7% (Fig 4, S3 Table). We found that *P*. *quinata* had a significantly lower belowground biomass fraction than *T*. *rosea* (p = 0.0032) or *D*. *retusa* (p = 0.0080) while *Anacardium excelsum*, *C*. *odorata*, and *T*. *amazonia* fell in between the highest and lowest groups. To assess the effect tree size on the %BGB relationship, we ran a beta regression model pooling the species. As BD increased, %BGB tended to decline (pseudo R^2^_adj_ = 0.06, χ^2^ = 2.6036, p < 0.0001) (Fig 5). However, while the relationship is significant, it is important to note that it is a very weak relationship.

#### Biomass of leaves, branches, stems, and roots

We found species differences related to allocation of biomass to roots, stems, branches, and leaves. Foliar (leaf) biomass ranged from 6% to 14% of total tree biomass (Fig 4). *Terminalia amazonia* had significantly greater foliar biomass allocation than either *C*. *odorata* (p = 0.0036) or *T*. *rosea* (p = 0.0101). Branch biomass allocation ranged from 27% to 40%. *Dalbergia retusa* allocated significantly more biomass to branches than *C*. *odorata* (p = 0.0217) or *T*. *rosea* (p = 0.440). Finally, stem biomass allocation ranged from 20% to 41% of the total tree (Fig 4). *Dalbergia retusa* had significantly less stem allocation than *C*. *odorata* (p = 0.0003), *P*. *quinata* (p = 0.0024), or *T*. *rosea* (p = 0.0244).

### Root architecture

We took three broad approaches to assess root architecture. The first was to examine the distances roots traveled horizontally. Here comparisons were made between root and crown radii. Root horizontal distances and depths were then compared among species. The second approach was to compare convex volume among species. Finally, we examined the allocation of biomass to a main root versus horizontal roots.

**Fig 6:**
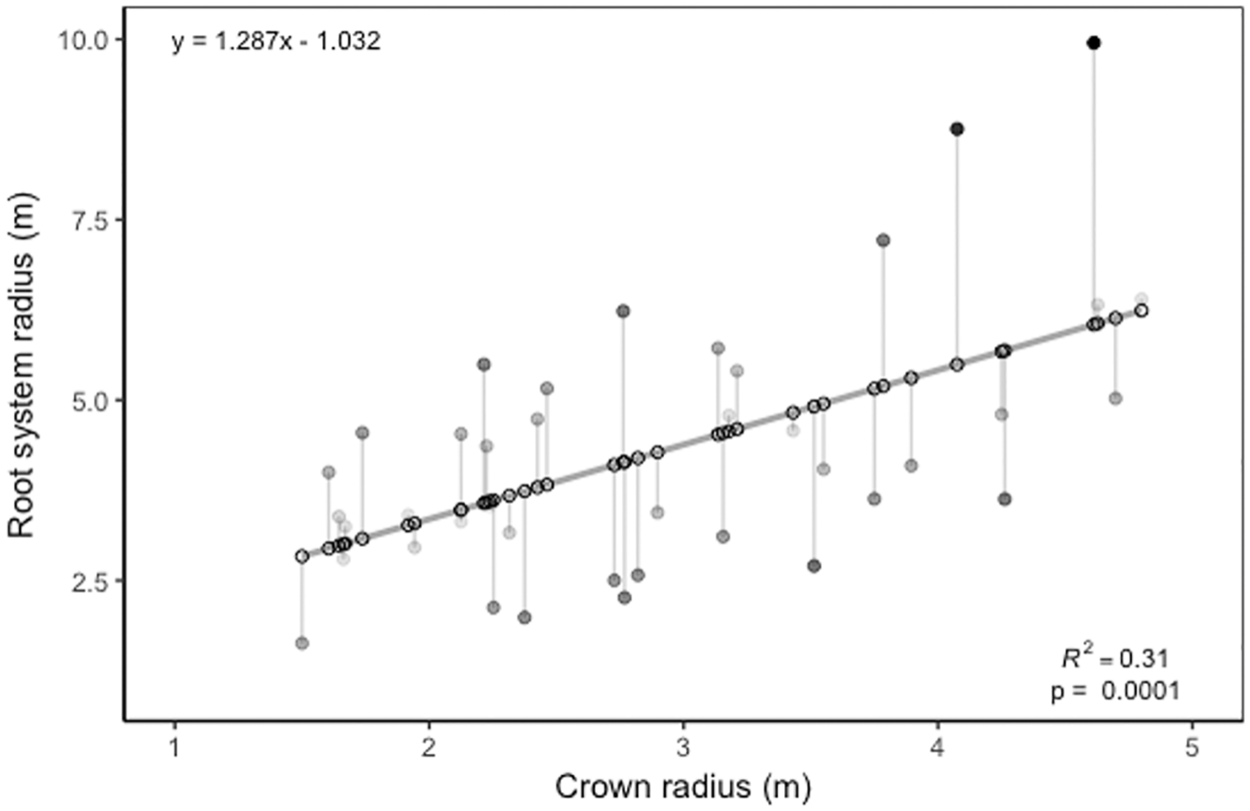
Crown radius (m) versus root system radius (m). Crown radius (m) of 6 study species plotted against maximum rooting distance (m) of 6 study species. Significant relationship exists between crown radius and root system radius (R^2^ = 0.31 p < 0.0001). Open circles represented modeled predicted values. Filled circles represent the residuals with darker circles being further from the predicted values than lighter, gray shaded circles. Gray line represents the linear model.

We found two of the 40 trees excavated (one individual of *Terminalia amazonia* and one of *Pachira quinata*) had coarse root grafts with neighboring trees of other species. This has been shown to be rare, but present in systems where soils are extremely saturated [62,63]. Mean horizontal rooting distance (i.e., root system radius (m)) from the bole of the tree was on average 1.28 times larger than mean crown radius (m) (pseudo R^2^ = 0.31, χ^2^ = 15.688, p = 0.0002) for the pooled species (Fig 6). We found that mean rooting radius was generally greater than mean crown radius and followed a significant linear relationship (R^2^ = 0.31, p < 0.0001). Interestingly, the maximum horizontal rooting distance nearly doubled that of the crown radius for some trees (S1 Fig; R^2^_adj_ = 0.29, p < 0.0002)). Important relationships and significant differences were found among species for the root architectural traits measured (S3 Table).

**Fig 7:**
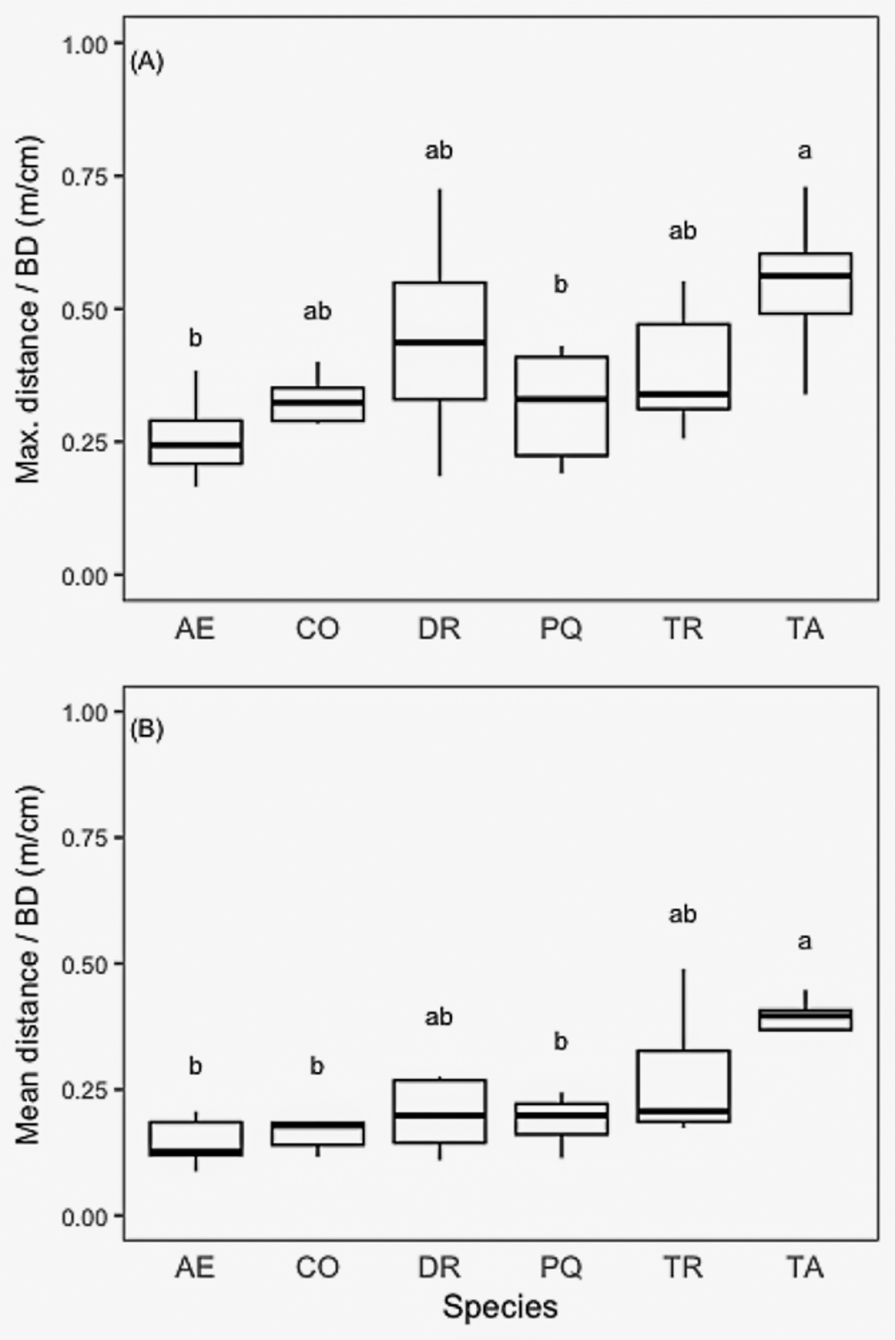
(A) Maximum horizontal root distance (m) and (B) mean horizontal root distance (m) by basal diameter (cm) for all six species. Letter denote significant differences among species. Axis letters signify the following: AE, *A*. *excelsum*, CO, *C*. *odorata*, DR, *D*. *retusa*, PQ, *P*. *quinata*, TR, *T*. *rosea*, TA, *T*. *amazonia*.

**Fig 8:**
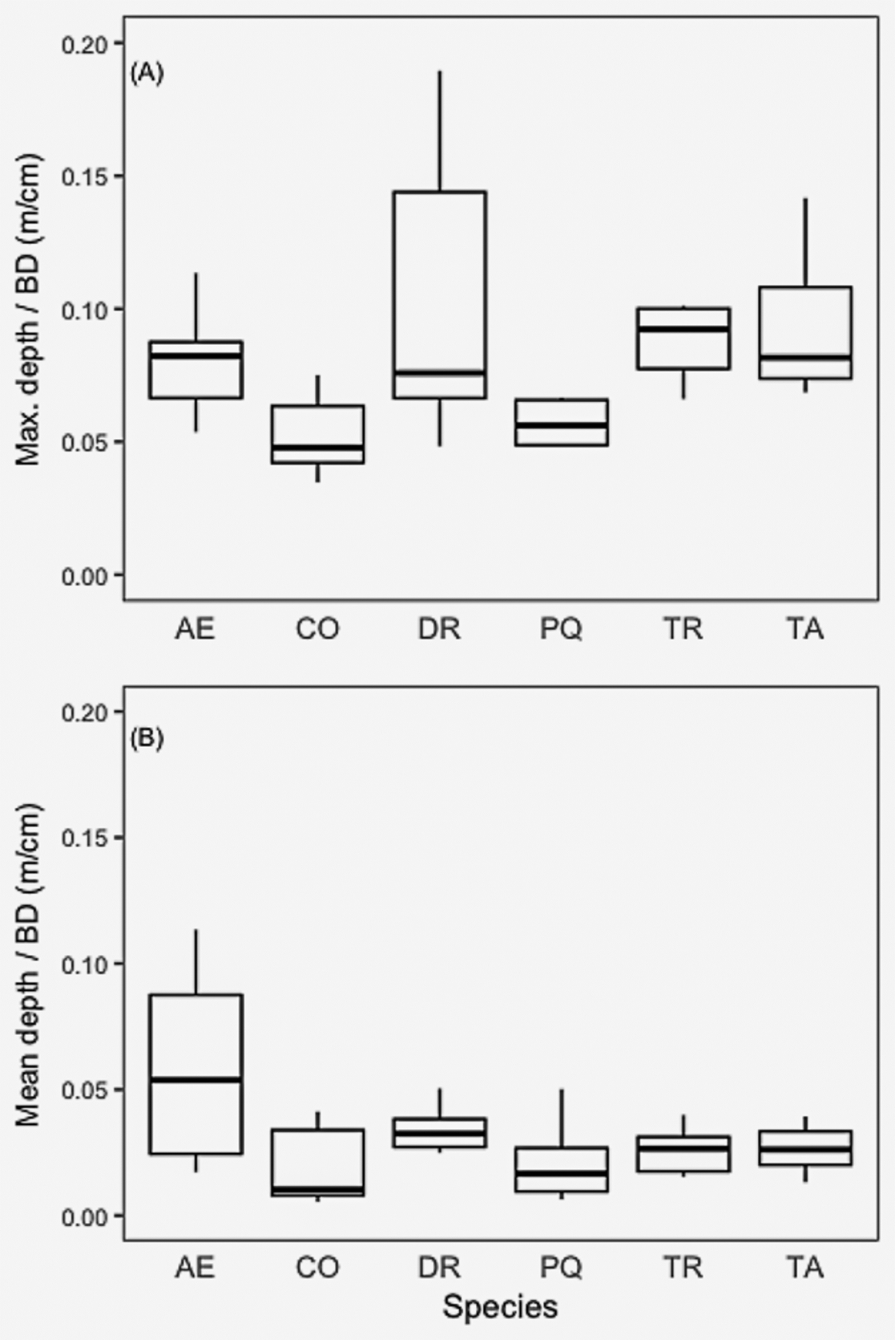
(A) Mean root depth and (B) maximum root depth by basal diameter of six study species. Axis letters signify the following: AE, *A*. *excelsum*, CO, *C*. *odorata*, DR, *D*. *retusa*, PQ, *P*. *quinata*, TR, *T*. *rosea*, TA, *T*. *amazonia*.

**Fig 9:**
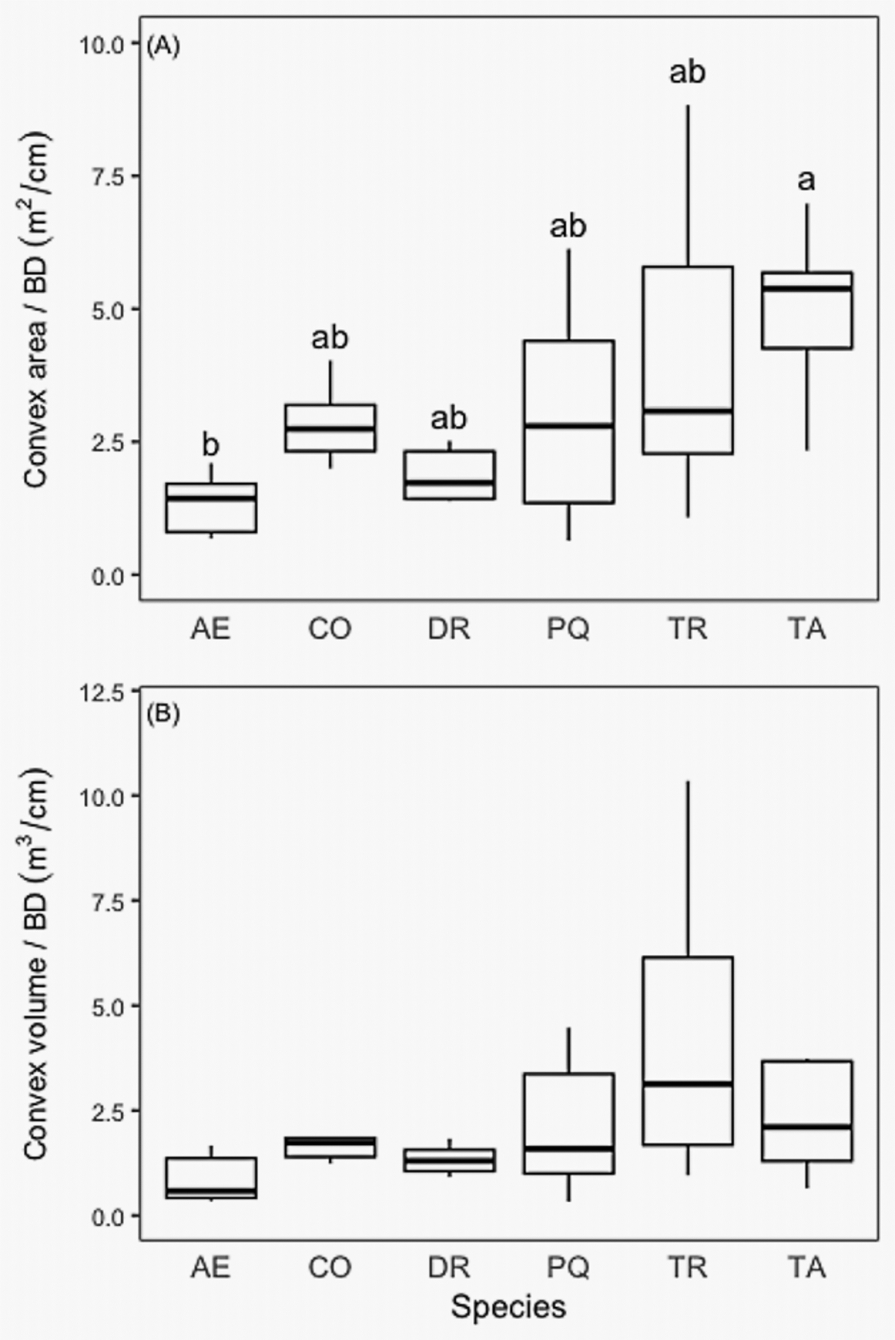
(A) Convex area (m^2^) and (B) convex volume (m^3^) by basal diameter (cm) of six study species. Axis letters signify the following: AE, *A*. *excelsum*, CO, *C*. *odorata*, DR, *D*. *retusa*, PQ, *P*. *quinata*, TR, *T*. *rosea*, TA, *T*. *amazonia*.

We calculated maximum and mean horizontal rooting distance for all six species scaled to BD. We found that *T*. *amazonia* had mean root distances significantly greater than *A*. *excelsum*, *C*. *odorata*, and *P*. *quinata* (Fig 7). We also compared maximum and mean rooting depth by basal diameter among species and found no significant differences among species for either maximum or mean depth of roots (Fig 8). While there were marked differences in root volume (Fig 9, S1 Table) the within-species variability was such that no significant differences were detected. Rooting volumes ranged from 1.0 m^3^ to 240.7 m^3^. The convex area of the species varied among species, following a similar pattern to rooting volume, with the exception of *T*. *amazonia* (Fig 9). When log-transformed, data showed no significant differences between species based on ANOVA and a post-hoc Tukey test (p < 0.05) (Fig 9).

**Fig 10:**
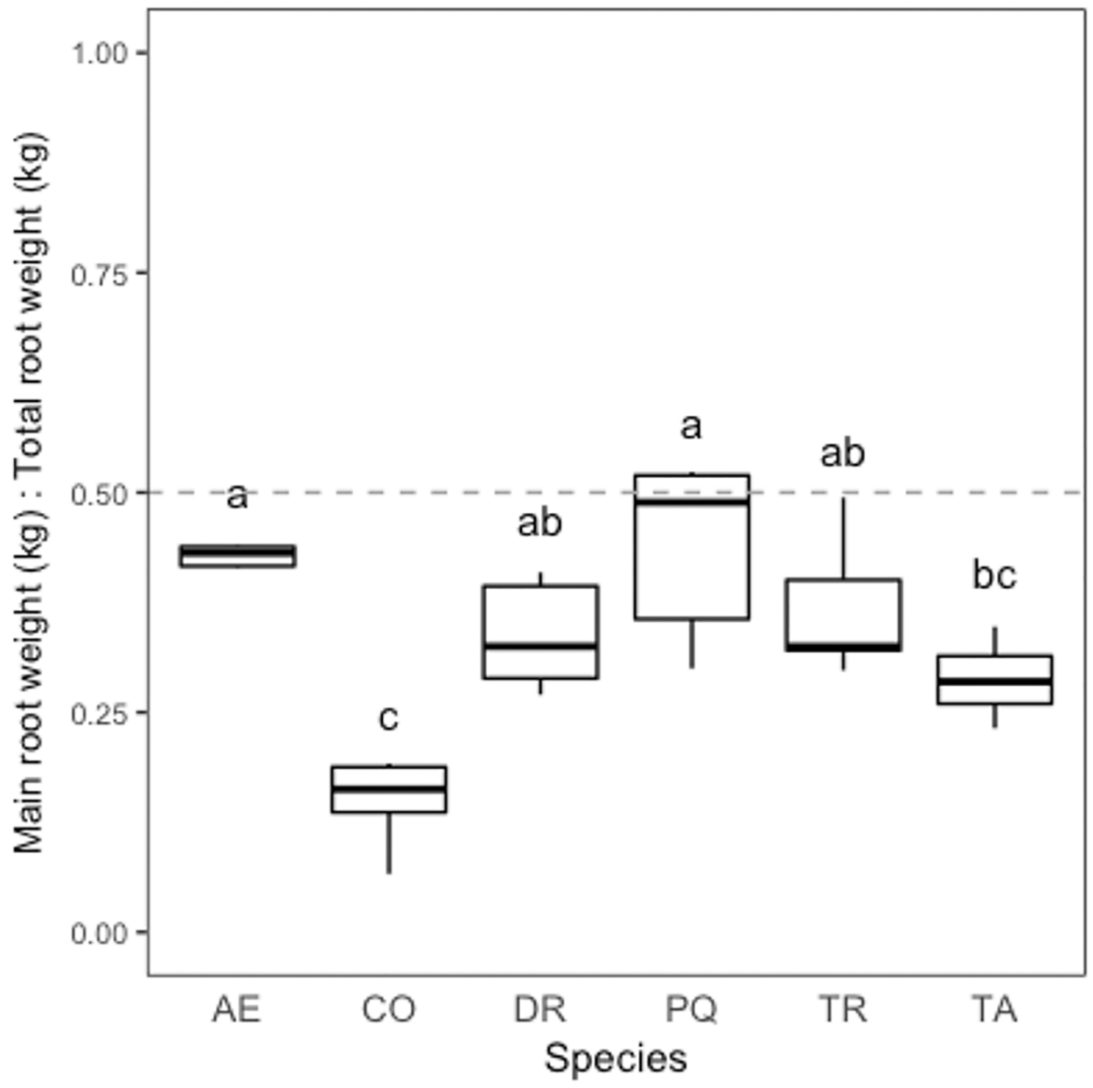
Main dry root weight to total dry root weight. Boxplot parameters in the figure are as follows: the horizontal line within the box visualizes the median, boxes comprise data between the 1^st^ and 3^rd^ quartile of the data, whiskers reach to 1.5x interquartile range added to/subtracted from the 3^rd^/1^st^ quartile. Axis letters signify the following: AE, *A*. *excelsum*, CO, *C*. *odorata*, DR, *D*. *retusa*, PQ, *P*. *quinata*, TR, *T*. *rosea*, TA, *T*. *amazonia*. Letters represent significant differences based on beta regression and likelihood ratio test.

Comparisons of main root mass to total root mass by species emphasize different allocation strategies by species. Our results show that root allocation differences were significant among species (pseudo R-squared = 0.62, χ^2^–38.258, p < 0.0001). For example, both *A*. *excelsum* and *P*. *quinata* allocate significantly more resources to a main root than *C*. *odorata* or *T*. *amazonia*, which allocate more resources to lateral roots. Both *D*. *retusa* and *T*. *rosea* fell between these two groups (Fig 10, S1 Table).

## Discussion

Multi-species equations to estimate forest biomass and species-specific equations to estimate plantation biomass are both pivotal links and major sources of uncertainty for estimating carbon stocks and accumulation rates at the plot and landscape level [37,38,64,65]. Direct measures of biomass obtained through destructive harvest, however, often do not include root systems even though root biomass can contribute greater than 25% of total tree biomass and provide insights into the functional role roots play in water cycling. Our study evaluated belowground and aboveground biomass of native species planted in the tropics. Unsurprisingly, BD and DBH were the strongest predictors of total biomass when compared to height, BD*height, DBH*height [32,34,66]. We also show AGB can predict TB, a potentially important finding for scaling AGB estimates of forests to TB and C stock estimates (Fig 3).

The range of values for total biomass (above- and below-ground) observed in this study can be attributed to the diversity of species and range of basal diameters (Fig 2). For example, *C*. *odorata* generally had the largest BD’s and had some of the highest total biomass values, a trend we expected to find based on work from Cole and Ewel 2006 [34]. *Pachira quinata* had BD’s close to the mean for our study, but accounted for some of the lowest total biomass values, likely attributable to the low wood specific gravity (WSG) of the species [67]. However, as shown in *Bombacopsis quinata* grown in Costa Rica, the biomass of this species begins to accumulate rapidly after year fifteen [68]. We expect that older the *P*. *quinata* may behave similarly.

### Species-specific aboveground and belowground biomass

In general, we found that allocation patterns to the belowground and aboveground compartments were similar across species (Fig 4). An average of 26.7% of total biomass was allocated to the roots while an average of 73.3% was allocated to the aboveground components. This biomass fraction closely resembles biomass fraction calculations from other studies that directly harvested tree roots [34,35]. Even though we used a direct method to estimate BGB, we underestimated BGB as we excluded fine roots. In a review of available studies, Vogt et al. [69] found that fine root biomass comprised of 8%, 1–2%, and 7–14% of the total biomass for broadleaf deciduous, broadleaf semi deciduous, and broadleaf evergreen trees, respectively. Future studies can address this underestimation by subsampling for fine roots [70] and by combining direct methods with indirect methods. Additional core samples would be necessary to then extrapolate fine root mass per soil volume.

### Allocation of biomass to leaves, branches, stems, and roots

Stem or branch biomass accounted for upwards of 40% of total biomass, on average (Fig 4). However, this varied by species, and may be attributed to the growth characteristics of individual species. For example, *D*. *retusa* has many branches and few stems, explaining the greater proportion of biomass allocated to branches. In contrast, *C*. *odorata*, allocated more to the stem than the branches, underlying the differences in growth patterns. Crown rise due to inter- or intraspecific shading can have a substantial impact on crown architecture and biomass relationships [54]. Yet our trees were not yet experiencing crown competition even though they were already experiencing competition belowground, meaning crown architecture likely had more to do with species differences than aboveground competition. As stands age, however, crown competition can have a greater role in branch and crown size and shape.

### Root architecture

Our work on root architecture (rooting depth, distance, area, and volume) provides insights into the hidden world of roots. Despite the trees not yet experiencing aboveground competition, the rooting zones of many trees overlapped and two individuals grafted coarse roots with neighbors. In just six species within the same guild, we show variability of rooting strategies and challenge the notion that root architecture will closely resemble the shape of the crown [55]. The maximum root system radius was nearly double that of maximum crown radii. These data underscore the fact that roots travel far beyond their tree crowns in terms of horizontal distance, even in moist tropical forests. The significance and strength of the relationship (Fig 6) can help guide ecological and modeling studies where knowing how far roots travel is important [44–49].

Perhaps the most interesting finding from our analyses is that species within the same guild [52] and grown in similar conditions have such a diversity of rooting patterns and architectures. Coll et. al., [35] worked at one of the same sites we did, finding differences in root architecture between successional guilds of species that were three years of age. Our study goes a step further and shows that there were structural differences even within the same guild, exhibiting an underappreciated axis in trait variation.

The two species with the highest relative biomass allocation to a main root (*A*. *excelsum* and *P*. *quinata*) had significantly higher allocation than *T*. *amazonia*, and are the same two species with significantly lower maximum and mean rooting distances than *T*. *amazonia*. *C*. *odorato* had significantly lower relative biomass allocation to a main root as compared to all others except *T*. *amazonia*. Taken together the horizontal root distance and relative main root biomass allocation clearly separate *T*. *amazonia* from *A*. *excelsum* and *P*. *quinata*. Although not significantly different from either of these groups for these attributes, the actual means for the main root of *D*. *retusa* and *T*. *rosea* are much closer to *T*. *amazonia* than the other group. While significant differences were found between some species in terms of relative biomass allocation to the main root, no differences were found between species for either maximum or mean rooting depth. This confirms field observations during excavations that species sending roots horizontally, or allocating more biomass to lateral roots than to main root, still send small coarse roots to deep soil horizons. Trees with pronounced main roots tend to break or snap off aboveground during wind storms while those with more pronounced lateral roots tend to blow over at the root zone and form root mounds [71]. Thus, while biomass allocation strategy may indicate mechanical differences, species allocating proportionally more biomass to lateral roots are not necessarily trading off the ability to forage for water and nutrients at great depths (Figs 7 & 8).

We found that crown architecture does not necessarily predict root architecture. For example, *A*. *excelsum* and *P*. *quinata* allocated more resources to a main root than the other lateral roots. Despite similar root architecture, these two species have very different aboveground patterns. For example, *A*. *excelsum* appears strongly excurrent aboveground (having a central leader) while *P*. *quinata* appears decurrent aboveground (having multiple scaffold branches). We also found variable below and aboveground shape for *T*. *amazonia* and *C*. *odorata*, both of which allocate more resources to lateral roots than a main root (Fig 10). Interestingly, *T*. *amazonia* has a somewhat Christmas-tree like structure aboveground in plantations while *C*. *odorata* has a rounded crown. Thus, our data show marked variability in rooting shape between species and irrespective of crown architecture within a uniform plantation setting. In forests or more diverse systems, structure and function might be even more diverse and difficult to predict [72].

## Conclusions

Carbon sequestration estimates necessitate more accurate allometric equations that estimate both below- and above-ground biomass. Here, we present species-specific allometric models for six tree species native to Panama and the Neotropics and a multi-species model using WSG.

We challenge previous notions about biomass allocation and rooting structure and suggest that the world of roots is, perhaps unsurprisingly, complicated. Not only did we find distinct root shapes belowground that did not always conform to the aboveground shape, but that roots begin competing well before branches of neighboring crowns. In plantations and forested systems, the root system within the soil profile plays a crucial role in nutrient and water cycling. Here we present data, summarize root shapes, and lend insight into the elusive belowground compartment. A better understanding of root architecture is necessary to understanding root function in terms of mechanical stability, resource acquisition, and ecosystem function.

## Supporting information

S1 Table Species-specific summary statistics (mean and standard error) for root calculations. Biomass fraction (root biomass), stem biomass, branch biomass, and foliar biomass mean and standard error for species. Mean and standard error for root and crown radii calculations, root distance, root depth, main:total root weight ratio, root volume, and total biomass calculations.
(TIF)

S2 Table Allometric equations for foliar, branch, and stem biomass. Foliar biomass (FB): models for foliar biomass. Branch biomass (BB): models to predict branch biomass. Stem biomass (SB): models to predict stem biomass. Equations use DBH (diameter at breast height, in cm), BD (basal diameter, in cm), H (height, m), DBH^2^*H, and BD^2^*H to predict biomass. Models: ‘*a*’ and ‘*b*’, coefficients for the species-specific allometric regression models in ln(y) = a + b x ln(x), where y is either FB, BB, or SB and x is either DBH or BD. R^2^, the adjusted R^2^; RMSE, root mean squared error; AICc, the second-order Akaike’s information criterion.
(TIF)

S3 Table Allometric equations for aboveground, belowground biomass, and total biomass. Aboveground biomass (AGB): models for aboveground biomass. Belowground biomass (BGB): models to predict belowground biomass. Total biomass (TB): models to predict total biomass. Equations use DBH (diameter at breast height, in cm), BD (basal diameter, in cm), H (height, m), DBH^2^*H, and BD^2^*H to predict biomass. Models: ‘*a*’ and ‘*b*’, coefficients for the species-specific allometric regression models in ln(y) = a + b x ln(x), where y is either FB, BB, or SB and x is either DBH or BD. R^2^, the adjusted R^2^; RMSE, root mean squared error; AICc, the second-order Akaike’s information criterion.
(TIF)

S4 Table Raw data of root and crown measurements taken in the field. This table includes all relevant raw data for DBH, BD, rooting depth, rooting distance, rooting area, crown diameter, and rooting volume for each of the study trees.
(TIF)

S5 Table Raw data for biomass calculations. This table includes all relevant data of height, BD, DBH, total biomass, aboveground biomass, belowground biomass, stem biomass, branch biomass, foliar biomass, and wood specific gravity for each study species.
(TIF)

S1 Fig Figure of crown radius (m) and maximum horizontal rooting distance (m). Pooled species show significant relationship between crown radius and maximum horizontal rooting distance based on linear regression and maximum likelihood estimator.
(TIF)

## Abbreviations

AGB: aboveground biomass
BB: branch biomass
BGB: belowground biomass
FB: foliar biomass
SB: stem biomass
TB: total biomass
WSG: wood specific gravity

## References

[1] Nair PKR. An introduction to agroforestry. Springer Science & Business Media; 1993.

[2] Ibrahim M, Villanueva C, Casasola F. Sistemas silvopastoriles como una herramienta para el mejoramiento de la productividad y rehabilitación ecológica de paisajes ganaderos en Centro América. 2007;

[3] Murgueitio E, Calle Z, Uribe F, Calle A, Solorio B. Native trees and shrubs for the productive rehabilitation of tropical cattle ranching lands. For Ecol Manage. 2011;261(10):1654–63.

[4] Tucker NIJ, Murphy TM. The effects of ecological rehabilitation on vegetation recruitment: some observations from the Wet Tropics of North Queensland. For Ecol Manage. 1997;99(1):133–52.

[5] Rodrigues RR, Gandolfi S, Nave AG, Aronson J, Barreto TE, Vidal CY, et al Large-scale ecological restoration of high-diversity tropical forests in SE Brazil. For Ecol Manage. 2011;261(10):1605–13.

[6] Piotto D, Craven D, Montagnini F, Alice F. Silvicultural and economic aspects of pure and mixed native tree species plantations on degraded pasturelands in humid costa rica. New For. 2010;39(3):369–85.

[7] Griess VC, Knoke T. Can native tree species plantations in Panama compete with Teak plantations? An economic estimation. New For. 2011;41(1):13–39.

[8] Ashton MS, Mendelsohn R, Singhakumara BMP, Gunatilleke CVS, Gunatilleke I, Evans A. A financial analysis of rain forest silviculture in southwestern Sri Lanka. For Ecol Manage. 2001;154(3):431–41.

[9] Montagnini F, Nair PKR. Carbon sequestration: an underexploited environmental benefit of agroforestry systems. Agrofor Syst. 2004;61(1–3):281–95.

[10] Locatelli B, Vignola R. Managing watershed services of tropical forests and plantations: can meta-analyses help? For Ecol Manage. 2009;258(9):1864–70.

[11] Chazdon RL. Beyond Deforestation: Restoring Forests and Ecosystem Services on Degraded Lands. Science (80-) [Internet]. 2008;320:1458–60. Available from: http://www.ncbi.nlm.nih.gov/pubmed/18556551 10.1126/science.1155365 18556551

[12] Jackson ST, Hobbs RJ. Ecological Restoration in the Light of Ecological History. Science (80-) [Internet]. 2009 7 30;325(5940):567–9. Available from: http://science.sciencemag.org/content/325/5940/567.abstract 10.1126/science.1172977 19644108

[13] Paquette A, Messier C. The role of plantations in managing the world’s forests in the Anthropocene. Front Ecol Environ. 2010;8:27–34.

[14] Hall JS, Ashton MS, Garen EJ, Jose S. The ecology and ecosystem services of native trees: Implications for reforestation and land restoration in Mesoamerica. For Ecol Manage [Internet]. 2011 5 [cited 2014 Mar 21];261(10):1553–7. Available from: http://linkinghub.elsevier.com/retrieve/pii/S0378112710007115

[15] Montagnini F, Porras C. Evaluating the Role of Plantations as Carbon Sinks: An Example of an Integrative Approach from the Humid Tropics. Environ Manage [Internet]. 1998;22(3):459–70. Available from: http://dx.doi.org/10.1007/s002679900119 9516537 10.1007/s002679900119

[16] Redondo-Brenes A. Growth, carbon sequestration, and management of native tree plantations in humid regions of Costa Rica. New For. 2007;34(3):253–68.

[17] Potvin C, Mancilla Lady, Buchmann N, Monteza J, Moore T, Murphy M, et al An ecosystem approach to biodiversity effects: Carbon pools in a tropical tree plantation. For Ecol Manage. 2011;261(10):1614–24.

[18] Holl KD, Zahawi RA. Factors explaining variability in woody above-ground biomass accumulation in restored tropical forest. For Ecol Manage. 2014;319:36–43.

[19] Moser G, Leuschner C, Hertel D, Graefe S, Soethe N, Iost S. Elevation effects on the carbon budget of tropical mountain forests (S Ecuador): The role of the belowground compartment. Glob Chang Biol. 2011;17:2211–26.

[20] Malhi Y, Doughty C, Galbraith D. The allocation of ecosystem net primary productivity in tropical forests. Philos Trans R Soc B Biol Sci. 2011;366(1582):3225–45. 10.1098/rstb.2011.0062 PMC3179639 22006964

[21] Asner GP, Mascaro J, Anderson C, Knapp DE, Martin RE, Kennedy-Bowdoin T, et al High-fidelity national carbon mapping for resource management and REDD+. Carbon Balance Manag [Internet]. 2013;8(1):7 Available from: http://www.pubmedcentral.nih.gov/articlerender.fcgi?artid=3717137&tool=pmcentrez&rendertype=abstract 23866822 10.1186/1750-0680-8-7 PMC3717137

[22] Drake JB, Knox RG, Dubayah RO, Clark DB, Condit R, Blair JB, et al Above-ground biomass estimation in closed canopy Neotropical forests using lidar remote sensing: Factors affecting the generality of relationships. Glob Ecol Biogeogr. 2003;12(2):147–59.

[23] Popescu SC, Wynne RH, Nelson RF. Measuring individual tree crown diameter with lidar and assessing its influence on estimating forest volume and biomass. Can J Remote Sens. 2003;29(5):564–77.

[24] Vazirabad YF, Karslioglu MO. Lidar for Biomass Estimation [Internet]. Biomass–Detection, Production and Usage. 2011 3–26 p. http://cdn.intechopen.com/pdfs/19065/InTech-Lidar_for_biomass_estimation.pdf

[25] Donoghue D, Watt P, Cox N, Wilson J. Remote sensing of species mixtures in conifer plantations using LiDAR height and intensity data. Remote Sens Environ [Internet]. 2007 10 30 [cited 2016 Mar 16];110(4):509–22. Available from: http://www.sciencedirect.com/science/article/pii/S0034425707001812

[26] Asner G, Palace M, Keller M, Pereira R. Estimating Canopy Structure in an Amazon Forest from Laser Range Finder and IKONOS Satellite Observations 1. Biotropica [Internet]. 2002 [cited 2014 Mar 13];34(4):483–92. Available from: http://onlinelibrary.wiley.com/doi/10.1111/j.1744-7429.2002.tb00568.x/abstract

[27] Ketterings QM, Coe R, Van Noordwijk M, Ambagau Y, Palm CA. Reducing uncertain in the use of allometric biomass equation for predciting above-ground tree biomass in mixed secondary forests. For Ecol Manage. 2001;146:199–209.

[28] Piotto D. A meta-analysis comparing tree growth in monocultures and mixed plantations. For Ecol Manage [Internet]. 2008 [cited 2013 Nov 4]; Available from: http://www.sciencedirect.com/science/article/pii/S0378112707007360

[29] Bauhus J, Khanna PK, Menden N. Aboveground and belowground interactions in mixed plantations of i<Eucalypus globulus>i and i<Acacia mearnsii>i. Can J For Res. 2000;30:1886–94.

[30] Yin C, Wang X, Duan B, Luo J, Li C. Early growth, dry matter allocation and water use efficiency of two sympatric Populus species as affected by water stress. Environ Exp Bot. 2005;53(3):315–22.

[31] van Breugel M, Ransijn J, Craven D, Bongers F, Hall JS. Estimating carbon stock in secondary forests: Decisions and uncertainties associated with allometric biomass models. For Ecol Manage [Internet]. 2011 10 [cited 2014 Jun 10];262(8):1648–57. Available from: http://linkinghub.elsevier.com/retrieve/pii/S0378112711004579

[32] Chave J, Andalo C, Brown S, Carins MA, Chambers JQ, Eamus D, et al Tree allometry and improved estimation of carbon stocks and balance in tropical forests. 2005;145:87–99. 10.1007/s00442-005-0100-x 15971085

[33] Cao J, Wang X, Tian Y, Wen Z, Zha T. Pattern of carbon allocation across three different stages of stand development of a Chinese pine (Pinus tabulaeformis) forest. Ecol Res. 2012;27:883–92.

[34] Cole TG, Ewel JJ. Allometric equations for four valuable tropical tree species. For Ecol Manage. 2006;229:351–60.

[35] Coll L, Potvin C, Messier C, Delagrange S. Root architecture and allocation patterns of eight native tropical species with different successional status used in open-grown mixed plantations in Panama. Trees—Struct Funct. 2008;22:585–96.

[36] Niiyama K, Kajimoto T, Matsuura Y, Yamashita T, Matsuo N, Yashiro Y, et al Estimation of root biomass based on excavation of individual root systems in a primary dipterocarp forest in Pasoh Forest Reserve, Peninsular Malaysia. J Trop Ecol. 2010;26:271.

[37] Pelletier J, Kirby KR, Potvin C. Significance of carbon stock uncertainties on emission reductions from deforestation and forest degradation in developing countries. For Policy Econ [Internet]. 2012;24:3–11. Available from: http://dx.doi.org/10.1016/j.forpol.2010.05.005

[38] Pelletier J, Ramankutty N, Potvin C. Diagnosing the uncertainty and detectability of emission reductions for REDD + under current capabilities: an example for Panama. Environ Res Lett. 2011;6(2):24005.

[39] Richardson AD, Statland CB, Gregoire TG. Root biomass distribution under three over types in a patchy Pseudotsuga menziesii forest in western Canada. Ann des Sci For. 2003;60(3):464–79.

[40] Kraenzel M, Castillo A, Moore T, Potvin C. Carbon storage of harvest-age teak (Tectona grandis) plantations, Panama. For Ecol Manage. 2003;173(2003):213–25.

[41] Fonseca W, Alice FE, Rey-Benayas JM. Carbon accumulation in aboveground and belowground biomass and soil of different age native forest plantations in the humid tropical lowlands of Costa Rica. New For. 2012;43:197–211.

[42] Kenzo T, Ichie T, Hattori D. Development of allometric relationships for accurate estimation of above-and below-ground biomass in tropical secondary forests in Sarawak, Malaysia. J Trop Ecol [Internet]. 2009 [cited 2013 Dec 9]; Available from: http://journals.cambridge.org/production/action/cjoGetFulltext?fulltextid=5645092

[43] Sah JP, Ross MS, Koptur S, Snyder JR. Estimating aboveground biomass of broadleaved woody plants in the understory of Florida Keys pine forests. For Ecol Manage. 2004;203(1–3):319–29.

[44] Dybzinski R, Farrior CE, Pacala SW. Increased forest carbon storage with increased atmospheric CO2 despite nitrogen limitation: A game-theoretic allocation model for trees in competition for nitrogen and light. Glob Chang Biol. 2015;21(3):1182–96. doi: 10.1111/gcb.12783 25392967 10.1111/gcb.12783

[45] Dybzinski R, Farrior C, Wolf A, Reich PB, Pacala SW. Evolutionarily stable strategy carbon allocation to foliage, wood, and fine roots in trees competing for light and nitrogen: an analytically tractable, individual-based model and quantitative comparisons to data. [Internet]. Vol. 177, The American naturalist. 2011 153–166 p. Available from: http://www.ncbi.nlm.nih.gov/pubmed/21460552 10.1086/657992 21460552

[46] Farrior CE, Rodriguez-Iturbe I, Dybzinski R, Levin SA, Pacala SW. Decreased water limitation under elevated CO2 amplifies potential for forest carbon sinks [Internet]. Vol. 112, Proceedings of the National Academy of Sciences. 2015 7213–7218 p. Available from: http://www.pnas.org/content/112/23/7213.abstract%5Cnhttp://www.pnas.org/content/112/23/7213.full.pdf 10.1073/pnas.1506262112 PMC4466696 26039985

[47] Jackson RB S HJ. the Global Biogeography of Roots. 2002;72(3):311–28.

[48] Wang Y, Xie Z, Jia B. Incorporation of a dynamic root distribution into CLM4.5: Evaluation of carbon and water fluxes over the Amazon. Adv Atmos Sci. 2016;33(9):1047–60.

[49] Zeng X. Global Vegetation Root Distribution for Land Modeling. J Hydrometeorol [Internet]. 2001;2(5):525–30. Available from: http://journals.ametsoc.org/doi/abs/10.1175/1525-7541(2001)002%3C0525:GVRDFL%3E2.0.CO%3B2%5Cnhttp://journals.ametsoc.org/doi/abs/10.1175/1525-7541%282001%29002%3C0525%3AGVRDFL%3E2.0.CO%3B2

[50] Mart L, Hall JS. Protocolo 2009 de Estudio de Excavación de Raíces en el Parque Nacional de Soberanía y Sardinilla (Colón). Antecedentes. 2009.

[51] Van Breugel M, Hall JS, Craven DJ, Gregoire TG, Park A, Dent DH, et al Early growth and survival of 49 tropical tree species across sites differing in soil fertility and rainfall in Panama. For Ecol Manage [Internet]. 2011 5 [cited 2014 Feb 4];261(10):1580–9. Available from: http://linkinghub.elsevier.com/retrieve/pii/S0378112710004780

[52] Ashton PMS, Hall JS. Chapter 12 The Ecology, Silviculture, and Use of Tropical Wet Forests with Special Emphasis on Timber Rich Types. In: Springer [Internet]. 2011 p. 145–91. http://www.ncbi.nlm.nih.gov/pubmed/15003161%5Cnhttp://cid.oxfordjournals.org/lookup/doi/10.1093/cid/cir991%5Cnhttp://www.scielo.cl/pdf/udecada/v15n26/art06.pdf%5Cnhttp://www.scopus.com/inward/record.url?eid=2-s2.0-84861150233&partnerID=tZOtx3y1%5Cnhttp://link.spr

[53] Paul C, Griess VC, Havardi-Burger N, Weber M. Timber-based agrisilviculture improves financial viability of hardwood plantations: A case study from Panama. Agrofor Syst. 2015;89(2):217–35.

[54] Mäkelä A, Valentine HT. Crown ratio influences allometric scaling in trees. Ecology. 2006;87(12):2967–72. 17249219 10.1890/0012-9658(2006)87[2967:criasi]2.0.co;2

[55] Cermák J, Riguzzi F, Ceulemans R. Scaling up from the individual tree to the stand level in Scots pine. I. Needle distribution, overall crown and root geometry. Ann des Sci For. 1998;55(1–2):63–88.

[56] Meinzer F, Goldstein G, Andrade J. Regulation of water flux through tropical forest canopy trees: Do universal rules apply? Tree Physiol [Internet]. 2001 [cited 2013 Dec 5]; Available from: http://treephys.oxfordjournals.org/content/21/1/19.short 10.1093/treephys/21.1.19 11260820

[57] Alvarado-Barrientos MS, Hernandez-Santana V, Asbjornsen H. Variability of the radial profile of sap velocity in Pinus patula from contrasting stands within the seasonal cloud forest zone of Veracruz, Mexico. Agric For … [Internet]. 2013 [cited 2013 Nov 12]; Available from: http://www.sciencedirect.com/science/article/pii/S0168192312002560

[58] Burgess SSO, Adams MA, Turner NC, Beverly CR, Ong CK, Khan AAH, et al An improved heat pulse method to measure low and reverse rates of sap flow in woody plants. Tree Physiol. 2001;21(9):589–98. 11390303 10.1093/treephys/21.9.589

[59] Bonnor GM. The influence of stand density on the correlation of stem diameter with crown width and height for lodgepole pine. For Chron. 1964;40(3):347–9.

[60] Dawkins HC. Crown Diameters: Their relation to bole diameter in tropical forest trees. Common Wealth For Rev. 1963;42(4):318–33.

[61] Farrari S, Cribari-Neto F. Beta Regression for Modelling Rates and Proportions. J Appl Stat. 2004;31(7):799–815.

[62] Keely JE. Population variation in root grafting and a hypothesis. Oikos. 1988;52(3):364–6.

[63] Bormann FH. The Structure, Function, and Ecological Significance of Root Grafts in Pinus strobus L. A. Ecol Monogr. 1966;36(1):1–26.

[64] FAO [Internet]. 2010 [cited 2015 Jan 7]. http://www.fao.org/docrep/012/i200e/0200e.pdf

[65] Hall JS, Aguilar-González B, Aguirre N, Asbjornsen H, Balvanera P, Berry ZC, et al Managing Watersheds for Ecosystem Services in the Steepland Neotropics [Internet]. 2015 Available from: http://publications.iadb.org/handle/11319/7233

[66] Ducey M, Zarin D, Vasconcelos S, Araújo M. Biomass equations for forest regrowth in the eastern Amazon using randomized branch sampling. Acta Amaz [Internet]. 2009 [cited 2013 Oct 31];39(2):349–60. Available from: http://www.scielo.br/scielo.php?pid=S0044-59672009000200013&script=sci_arttext

[67] Wishnie M, Dent D, Mariscal E, Deago J, Cedeno N, Ibarra D, et al Initial performation and reforestation potential of 24 tropical tree species planted across precipitation gradient in the Republic of Panama. For Ecol Manag. 2007;243:39–49.

[68] Pérez Cordero LD, Kanninen M. Wood specific gravity and aboveground biomass of Bombacopsis quinata plantations in Costa Rica. For Ecol Manage. 2002;165(1–3):1–9.

[69] Vogt KA., Vogt DJ., Palmiotto PA., Boon P, O’Hara J, Asbjornsen H. Review of root dynamics in forest ecosystems grouped by climate, climatic forest type and species. Plant Soil. 1996;187(2):159–219.

[70] Fonseca W, Alice FE, Rey-Benayas JM. Carbon accumulation in aboveground and belowground biomass and soil of different age native forest plantations in the humid tropical lowlands of Costa Rica. New For. 2012;43(2):197–211.

[71] Nicoll B, Ray D, Nicoll BC. Nicoll and Ray 1996 root architecture. full site conditions. 2015;(October).

[72] Markesteijn L, Poorter L. Seedling root morphology and biomass allocation of 62 tropical tree species in relation to drought- and shade-tolerance. J Ecol. 2009;97(2):311–25.

