# Supplementary figures and images for: Unearthing the hidden world of roots: Root biomass and architecture differ among species within the same guild
Source: PLoS One. 2017 Oct 12;12(10):e0185934. doi: 10.1371/journal.pone.0185934 (PMC5638295; doi:10.1371/journal.pone.0185934)

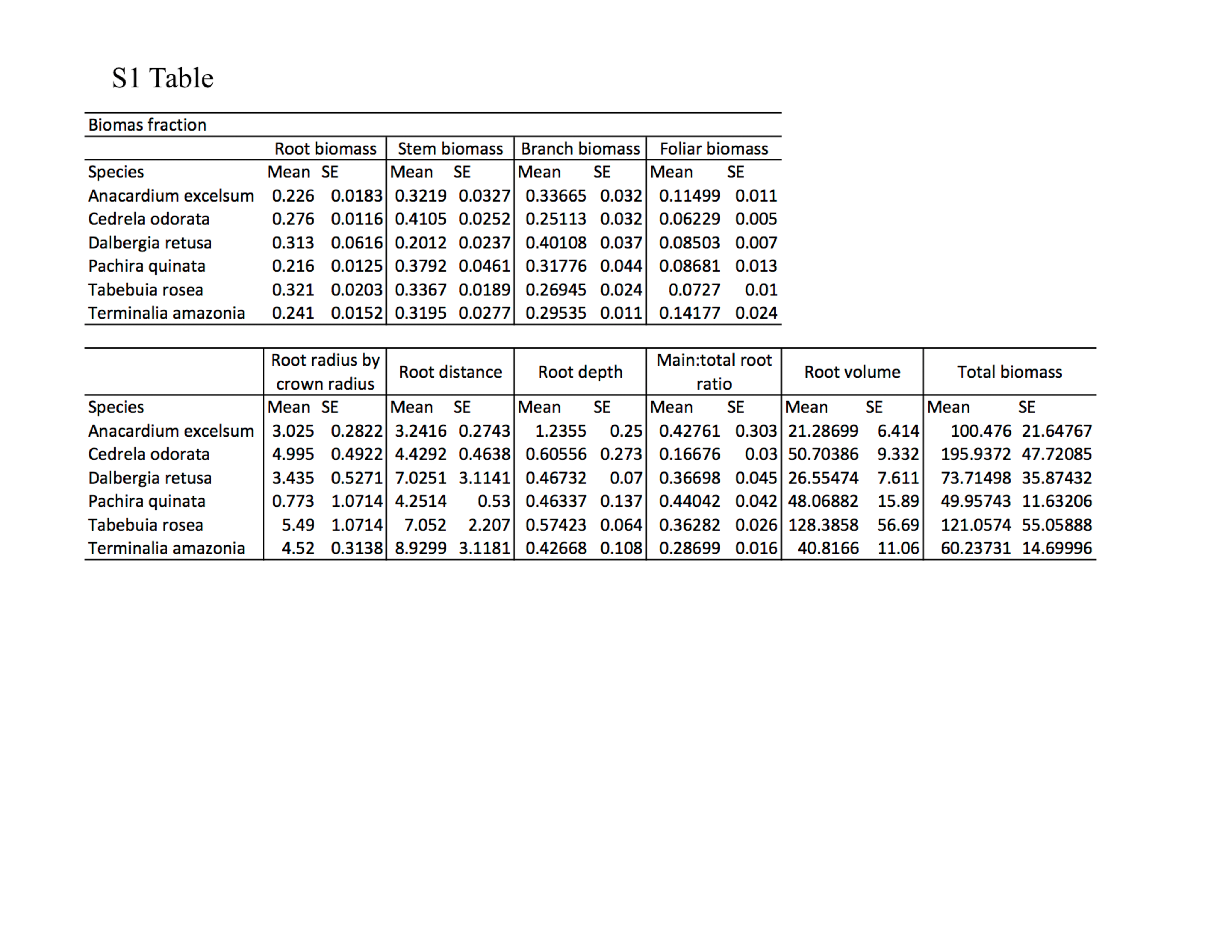

Supplement: S1 Table — Biomass fraction (root biomass), stem biomass, branch biomass, and foliar biomass mean and standard error for species. Mean and standard error for root and crown radii calculations, root distance, root depth, main:total root weight ratio, root volume, and total biomass calculations. (TIF) [file pone.0185934.s001.tif]

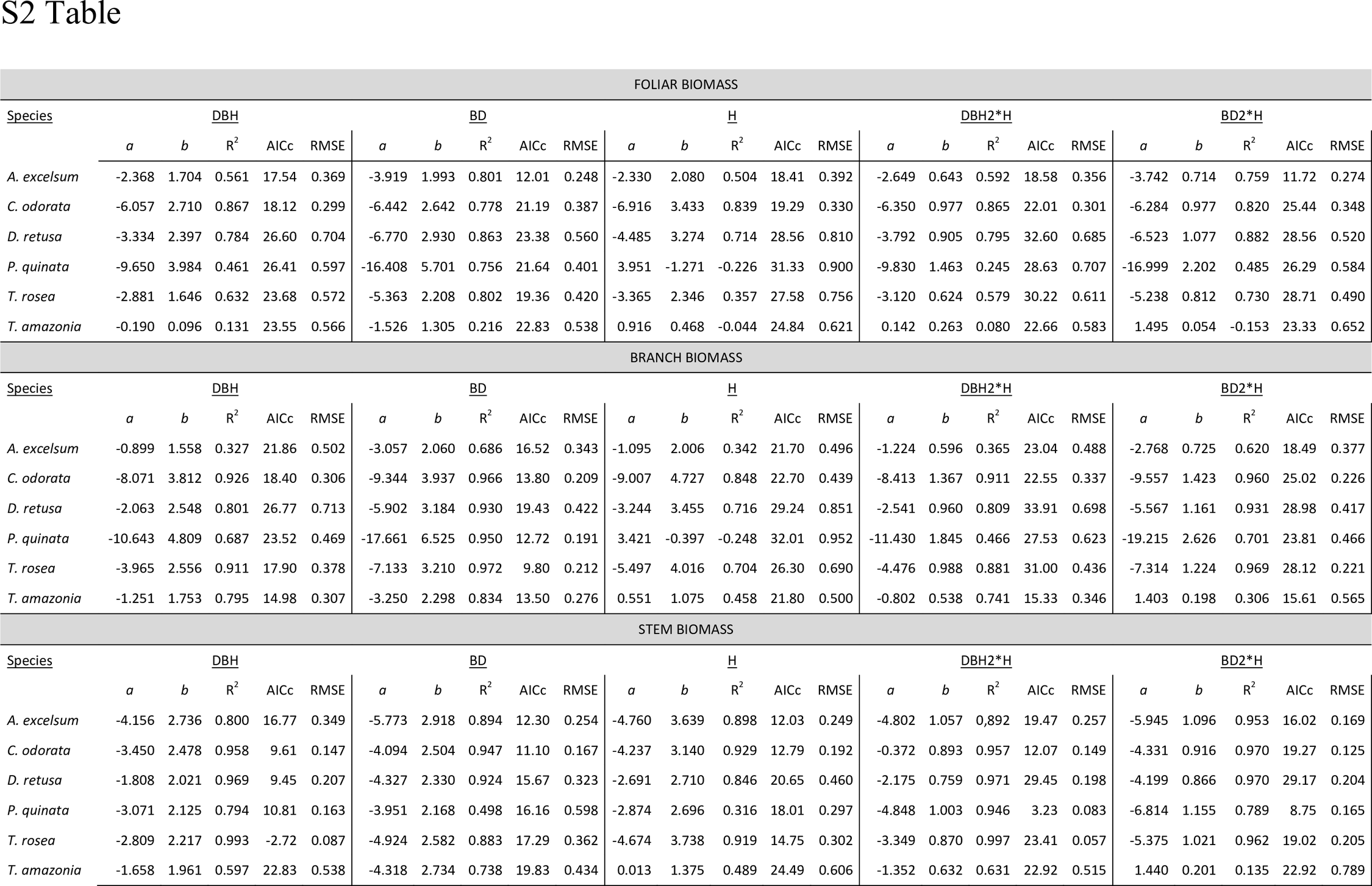

Supplement: S2 Table — Foliar biomass (FB): models for foliar biomass. Branch biomass (BB): models to predict branch biomass. Stem biomass (SB): models to predict stem biomass. Equations use DBH (diameter at breast height, in cm), BD (basal diameter, in cm), H (height, m), DBH2*H, and BD2*H to predict biomass. Models: ‘a’ and ‘b’, coefficients for the species-specific allometric regression models in ln(y) = a + b x ln(x), where y is either FB, BB, or SB and x is either DBH or BD. R2, the adjusted R2; RMSE, root mean squared error; AICc, the second-order Akaike’s information criterion. (TIF) [file pone.0185934.s002.tif]

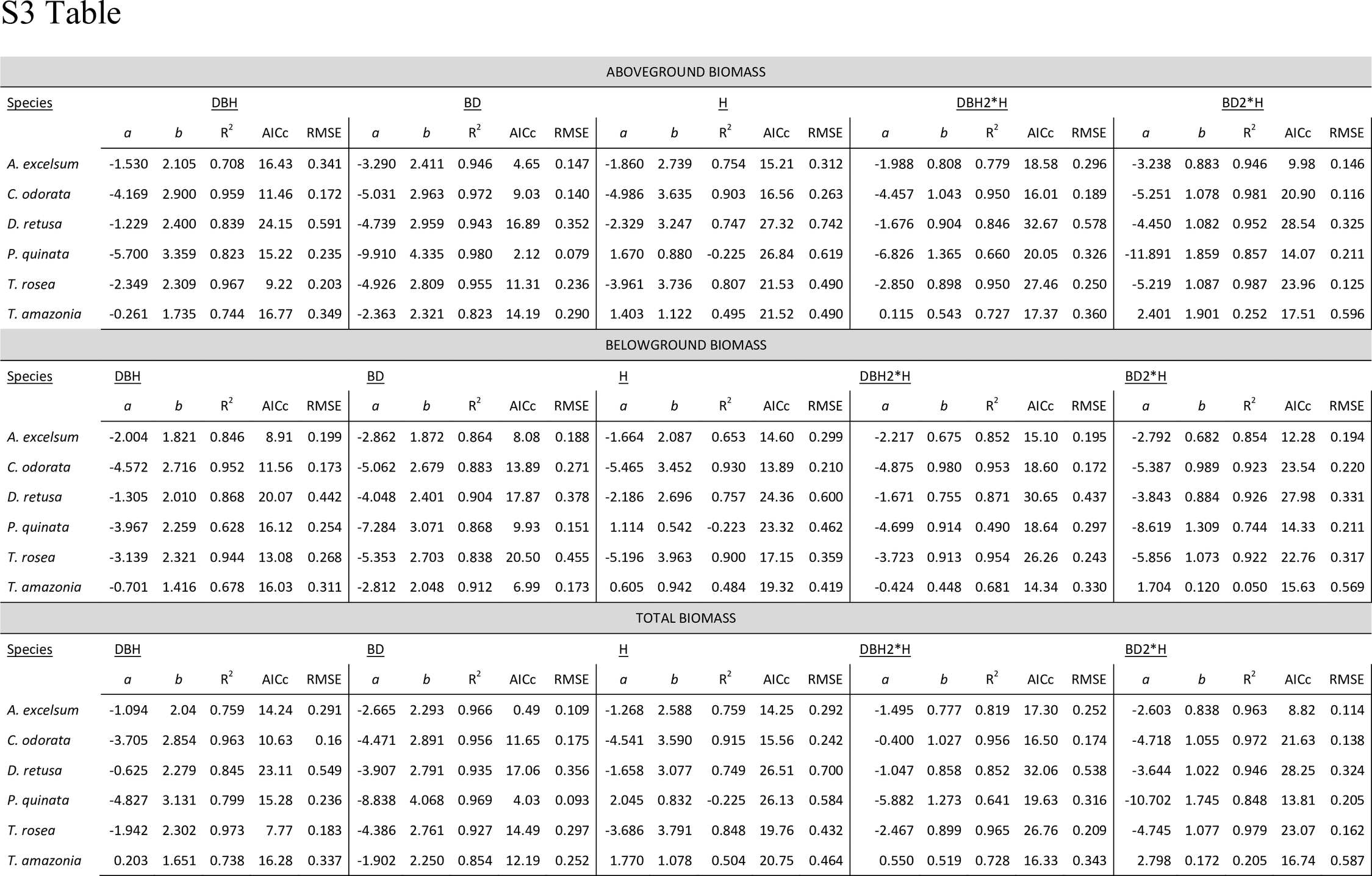

Supplement: S3 Table — Aboveground biomass (AGB): models for aboveground biomass. Belowground biomass (BGB): models to predict belowground biomass. Total biomass (TB): models to predict total biomass. Equations use DBH (diameter at breast height, in cm), BD (basal diameter, in cm), H (height, m), DBH2*H, and BD2*H to predict biomass. Models: ‘a’ and ‘b’, coefficients for the species-specific allometric regression models in ln(y) = a + b x ln(x), where y is either FB, BB, or SB and x is either DBH or BD. R2, the adjusted R2; RMSE, root mean squared error; AICc, the second-order Akaike’s information criterion. (TIF) [file pone.0185934.s003.tif]

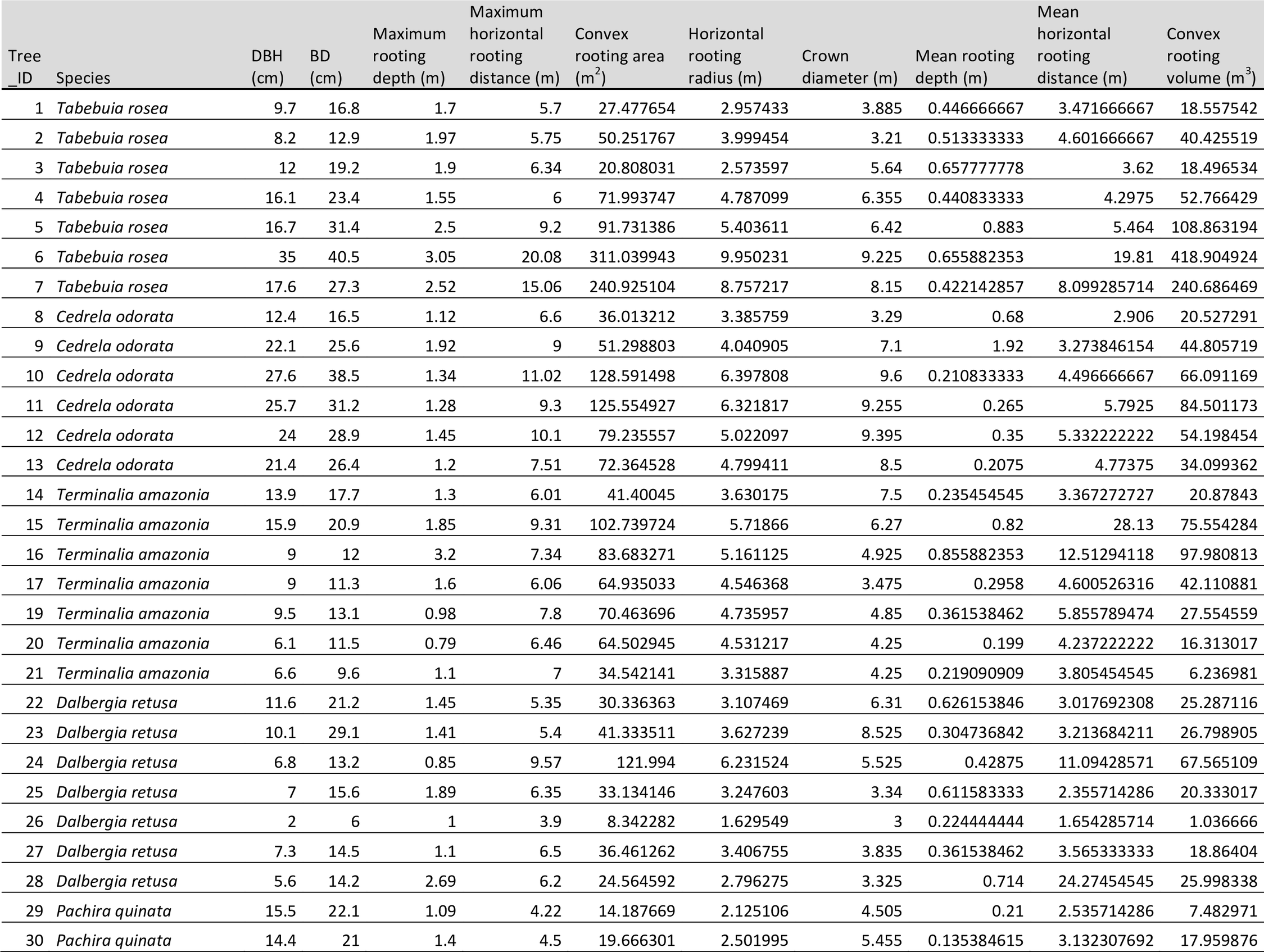

Supplement: S4 Table — This table includes all relevant raw data for DBH, BD, rooting depth, rooting distance, rooting area, crown diameter, and rooting volume for each of the study trees. (TIF) [file pone.0185934.s004.tif]

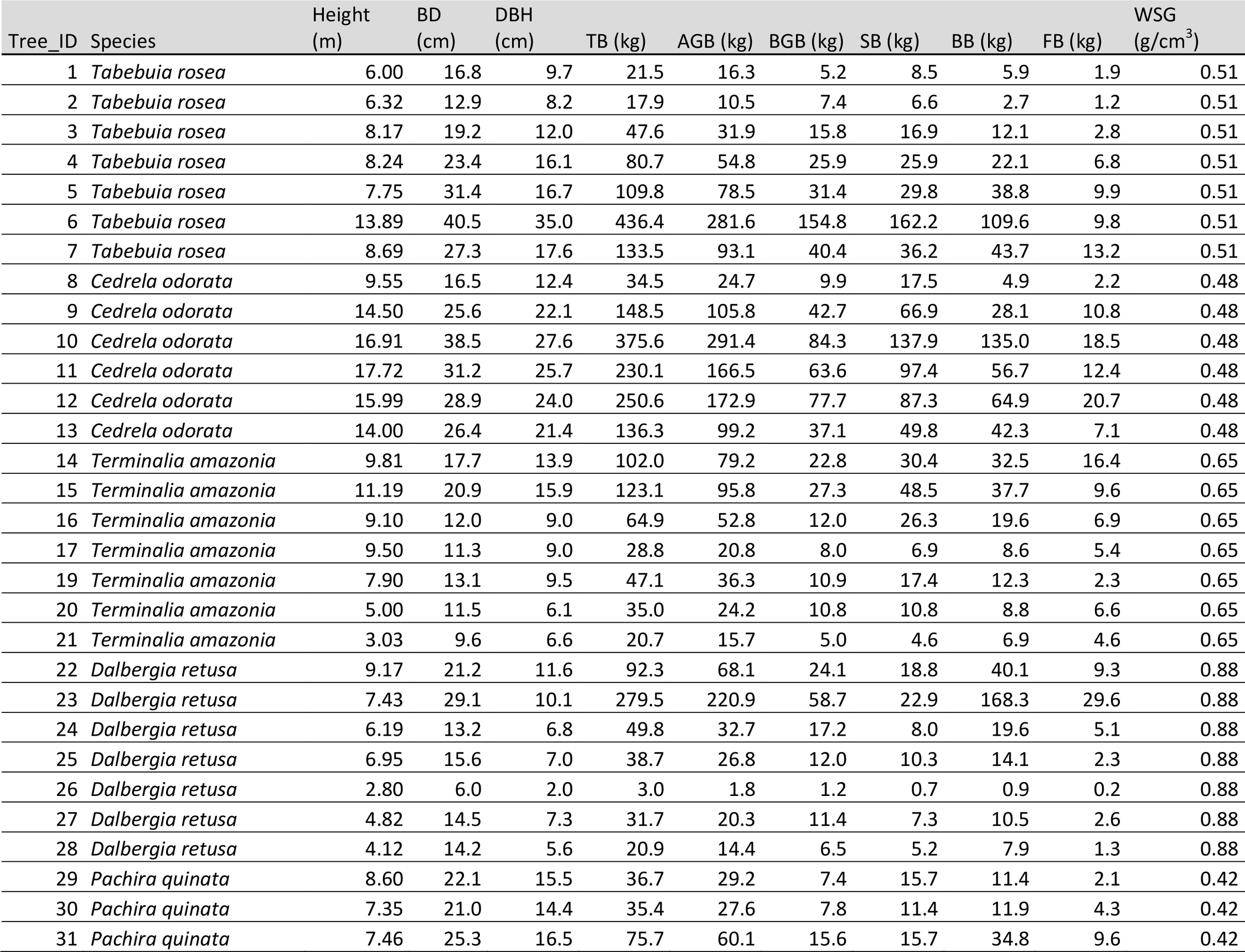

Supplement: S5 Table — This table includes all relevant data of height, BD, DBH, total biomass, aboveground biomass, belowground biomass, stem biomass, branch biomass, foliar biomass, and wood specific gravity for each study species. (TIF) [file pone.0185934.s005.tif]

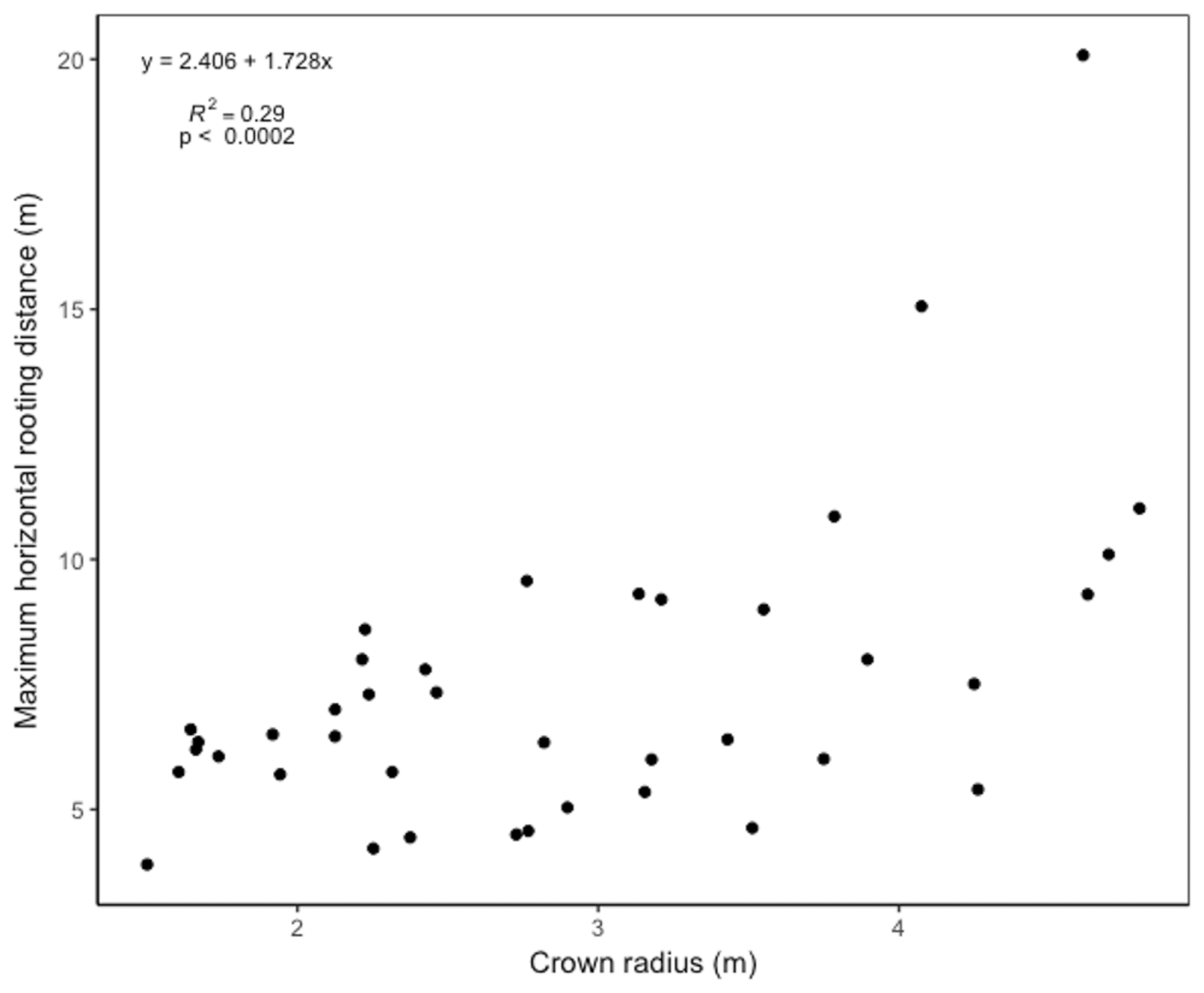

Supplement: S1 Fig — Pooled species show significant relationship between crown radius and maximum horizontal rooting distance based on linear regression and maximum likelihood estimator. (TIF) [file pone.0185934.s006.tif]
